# Redemption of Specific Categories of WIC Food Benefits and Risk of Program Discontinuation

**DOI:** 10.1001/jamanetworkopen.2025.46544

**Published:** 2025-12-03

**Authors:** M. Pia Chaparro, Shannon E. Whaley, Christopher E. Anderson

**Affiliations:** 1 Department of Health Systems and Population Health, School of Public Health, University of Washington, Seattle; 2 Food Systems, Nutrition, and Health Program, School of Public Health, University of Washington, Seattle; 3 Division of Research and Evaluation, Public Health Foundation Enterprises WIC, A Program of Heluna Health, City of Industry, California; 4 Department of Public Health, College of Education, Health, and Human Sciences, University of Tennessee, Knoxville

## Abstract

**Importance:**

Longer participation in the Special Supplemental Nutrition Program for Women, Infants, and Children (WIC) is positively associated with child diet quality; yet, child WIC participation decreases with age. A lower redemption rate for the WIC food package overall has been associated with risk of program discontinuation.

**Objective:**

To assess the association between redemption of specific WIC foods and risk of program discontinuation.

**Design, Setting, and Participants:**

This retrospective cohort study used WIC redemption data linked to administrative data from November 11, 2019, to June 30, 2023, and comprised a population-based sample of WIC participants from birth to 3 years of age in Southern California. Data were analyzed from May 22, 2024, to August 26, 2025.

**Exposure:**

Household-level redemption of WIC food benefit categories (fruits and vegetables, eggs, whole milk, cheese or tofu, 100% fruit or vegetable juice, reduced fat milk, legumes, canned fish, breakfast cereal, bread or whole grains, yogurt, infant formula, therapeutic infant formula, infant fruits and vegetables, infant cereal, and infant meats), averaged across certification periods and interval scaled in 10% increments.

**Main Outcomes and Measures:**

WIC program discontinuation was measured as a failure to complete eligibility recertification following the 12-month certification period (no [certified, any food benefit issuance in 2 months postcertification] compared with yes [discontinued, no food benefit issuance in 2 months postcertification]).

**Results:**

The sample included 188 368 participants (50.9% male; 46.9% aged 0-12 months at baseline; mean [SD], 1.5 years [1.2 years]). The most redeemed (≥70% redemption rate) WIC foods were infant formula (86.0%), fruits and vegetables (69.6%), eggs (67.1%), and whole milk (61.4%). Using covariate-adjusted generalized estimating equation Poisson regression, the study found that lower redemption (<70% vs 70%-100%) of fruits and vegetables, eggs, infant formula, or whole milk was associated with risk of higher program discontinuation, following a somewhat linear fashion, particularly among children younger than 2 years. For example, redeeming less than 10%, 10% to less than 20%, 20% to less than 30%, 30% to less than 40%, 40% to less than 50%, 50% to less than 60%, and 60% to less than 70% of WIC fruit and vegetable benefits was associated with a risk of program discontinuation that was 2.08 (95% CI, 2.01-2.14), 1.77 (95% CI, 1.71-1.83), 1.73 (95% CI, 1.67-1.78), 1.54 (95% CI, 1.50-1.59), 1.48 (95% CI, 1.44-1.52), 1.37 (95% CI, 1.33-1.41), and 1.24 (95% CI, 1.21-1.27) times higher, respectively, compared with redeeming 70% to 100% of fruit and vegetables issued benefits.

**Conclusions and Relevance:**

In this cohort study of WIC participants, findings suggest that understanding the redemption patterns of participants’ most highly valued foods could assist in identifying children at risk for program discontinuation, information that is crucial for maintaining WIC participation and maximizing positive nutritional outcomes of longer WIC participation.

## Key Points

**Question:**

Is redemption of specific foods from the Special Supplemental Nutrition Program for Women, Infants, and Children (WIC) associated with risk of program discontinuation?

**Findings:**

In this cohort study that included 188 368 WIC participants in Southern California, a lower redemption rate for fruits and vegetables, eggs, infant formula, and whole milk—the most highly redeemed WIC foods—was significantly associated with higher risk of program discontinuation.

**Meaning:**

These findings suggest that understanding the redemption patterns of participants’ most highly valued foods could assist in identifying young children at high risk for WIC program discontinuation.

## Teaser

This cohort study assesses the association between redemption of specific Women, Infants, and Children foods with risk of program discontinuation among participants in Southern California.

## Introduction

The Special Supplemental Nutrition Program for Women, Infants, and Children (WIC) is a federal nutrition assistance program serving pregnant and postpartum women and infants (defined as 0 to younger than 12 months) and children younger than 5 years who live in low-income households and are at nutritional risk. WIC provides supplemental healthy foods, nutrition education, breastfeeding support, and medical and social services referrals. In fiscal year 2023, approximately 6.5 million families participated in WIC, including 1.5 million infants aged 0 to 12 months and 3.6 million children aged 1 to 4 years,^1^ making it the third largest nutrition assistance program in the US.

Longer duration of WIC participation is associated with higher household food security,^2^ higher diet quality,^2,3,4^ and lower sugar-sweetened beverage consumption^5^ among children. Yet, many families stop participating in WIC while they are still income eligible, with the percentage of eligible infants and children participating in WIC decreasing from 78.0% at 0 to 12 months to 24.7% at 4 to 5 years.^6^ Lower redemption of WIC foods is associated with early program discontinuation.^7,8^ In particular, research in Southern California found that participants in households redeeming less than 70% of their benefits (vs 70%-100%) had a higher risk of program discontinuation, with significant increases in risk of discontinuation for each 10% decrease in benefit redemption.^7^

Research shows that some WIC foods are more favored by participants than others. In a study conducted in Oklahoma, the most redeemed WIC foods were infant formula (98% redemption rate), whole milk (83%), eggs (80%), juice (79%), and fruits and vegetables (79%), whereas the least redeemed were infant meats (37%).^9^ Similarly, studies in Massachusetts^10^ and California^11,12^ reported that infant formula and fruits and vegetables were the most redeemed WIC foods, and infant meats were the least redeemed. Research, both quantitative^13,14^ and qualitative,^15,16,17^ on the recent increase in the fruits and vegetables benefit also demonstrate the popularity of this benefit among WIC families.

What is currently unknown is whether redemption of specific WIC foods is associated with risk of WIC program discontinuation. Being able to identify families at risk of early discontinuation is a priority for WIC, to inform their retention efforts and maximize the nutritional impact of WIC participation. Among WIC participants aged 0 to 3 years in Southern California, the aims of this study were to assess (1) the association between redemption of specific WIC foods and risk of program discontinuation and (2) whether this association varied by age and participant type. We hypothesized that lower redemption of the most popular WIC foods would be associated with higher risk of program discontinuation.

## Methods

### Study Population

This cohort study used WIC administrative data, collected during routine service delivery by Public Health Foundation Enterprises WIC in Southern California. These data contain service dates (beginning and ending of certification periods), food benefit issuance and redemption, and participants’ sociodemographic characteristics. We used data from certification periods that began between November 11, 2019—the date when Public Health Foundation Enterprises WIC started transitioning to electronic benefit transfer issuance of WIC food benefits^18^—and June 30, 2022, for WIC participants younger than 4 years at the certification start date. Certification periods with some missing issuance (described in eFigure 1 in Supplement 1) were excluded from analysis (eMethods in Supplement 1), leaving a final analytical sample of 188 368 infants and children; 148 723 families; and 366 212 certification periods. eTable 1 in Supplement 1 shows characteristics of those included and excluded from the sample. The California Health and Human Services Agency Committee for the Protection of Human Subjects determined this study to be exempt from institutional review board review because deidentified data were used, and informed consent requirements were waived. This report was written to conform to the Strengthening the Reporting of Observational Studies in Epidemiology (STROBE) reporting guideline for cohort studies.

### Study Measures

Continued WIC participation is contingent on eligibility recertification, which, for this sample, occurred every 12 months. Discontinued WIC participation (ie, failure to complete eligibility recertification) following the index 12-month certification period was the outcome. Discontinuation of WIC participation was measured using the issuance of a food package to the WIC participant in the 2 months following the end of the index certification period (discontinued: no issuance; continued: any issuance). Further information on variable operationalization is available in the eMethods and eFigure 2 in Supplement 1.

Data on WIC food benefit redemption, the study’s exposure, were available from November 2019 to June 2023 at the household level because benefits for all household participants are issued and redeemed with a single electronic benefit transfer card. WIC food benefits are issued and redeemed in the following categories: fruits and vegetables, eggs, whole milk, cheese or tofu, 100% fruit or vegetable juice, reduced fat milk, legumes, canned fish, breakfast cereal, bread or whole grains, yogurt, infant formula, therapeutic infant formula, infant fruits and vegetables, infant cereal, and infant meats. Category-specific redemption percentages were calculated monthly by dividing the amount redeemed by the amount issued and multiplying by 100. Total WIC benefit redemption (across all categories of WIC foods) was calculated monthly as the average of all category-specific redemption percentages. Category-specific redemption and total WIC food benefit redemption were then averaged across all months within each full certification period, interval scaled in 10% increments, and categorized. This approach was chosen to account for nonmonotonic associations between redemption and recertification.

WIC administrative data available as covariates include age and participant category (0-12 months, formula fed; 0-12 months, partially breastfed; 0-12 months, fully breastfed; child 1 year; child 2 years; child 3 years), sex (male or female), and race and ethnicity; caregiver educational attainment (less than high school, completed high school, greater than high school) and language preference (Spanish, English, and other); and household size (<4 people, 4-5 people, ≥6 people), number of WIC participating individuals (1, 2, or ≥3), income as percentage of the federal poverty level (FPL; <50% FPL, 50 to <100% FPL, ≥100% FPL), Medicaid participation (yes or no), and Supplemental Nutrition Assistance Program (SNAP) participation (yes or no). Race was reported by the child’s caregiver from a list with specified options (eMethods in Supplement 1). Ethnicity (Hispanic, non-Hispanic) was presented as a separate question. Given small response frequencies for many specific racial categories, race and ethnicity were grouped into a single race and ethnicity variable by the researchers, with final categories including Hispanic (any race), non-Hispanic Asian, non-Hispanic Black, non-Hispanic other, and non-Hispanic White (grouping details in the eMethods in Supplement 1). Language preference, reported by the caregiver, was used to further divide the non-Hispanic Asian group into “English” and “other language” groups, and Hispanic into “English” and “Spanish” groups (final race and ethnicity and language categories: non-Hispanic Asian, English; non-Hispanic Asian, other; non-Hispanic Black; Hispanic, English; Hispanic, Spanish; non-Hispanic White; non-Hispanic other). Participants in all racial and ethnic categories could potentially speak a language other than English, but only non-Hispanic Asian and Hispanic groups reported other languages in enough numbers to allow for categorization.

Due to the study period including the COVID-19 pandemic, which brought several changes to WIC, variables were created indicating the number of months in the certification period that the household received an augmented WIC fruit and vegetable benefit and was exposed to WIC brand and package size flexibilities and/or WIC infant formula flexibilities (eMethods in Supplement 1). Missing benefit issuance has been associated with substantial differences in the risk of discontinued WIC participation^7^; thus, variables indicating missing issuance during the certification period were generated (any missing issuance, vs none, in the last 12, 6, or 3 months of the certification period).

### Statistical Analysis

Characteristics of study participants were summarized in categories of interval-scaled total WIC benefit redemption across certification periods, allowing for multiple observations per participant, using frequencies and percentages (categorical variables) or mean (SD) values (continuous variables). Distributions of each category-specific WIC benefit redemption variable were summarized with the frequency and percentage of certification periods for study participants in each interval-scaled category.

The association of category-specific WIC food benefit redemption with discontinued WIC participation was estimated using generalized estimating equation Poisson regression models with robust standard errors,^19^ accommodating clustering of multiple certification periods for individual participants and multiple participants within households, and including independent terms for interval-scaled redemption of the specific WIC food benefit category; the percentage of all WIC foods redeemed during the certification period; and child, caregiver, and household covariates. A sensitivity analysis, with identical parameters except for the exclusion of percentage of all WIC foods redeemed, was conducted. A second set of regression models, parameterized identically except for the addition of a 2-way interaction between interval-scaled redemption percentage of category-specific WIC food benefits and age and participant category, was run to assess whether associations were modified by age and category. Generalized estimating equation linear regression models, with identical independent and dependent variables to the 2-way interaction models, were run to estimate the proportion of participants discontinuing WIC participation by redemption rate and age and participant category. Additional Poisson regression models dichotomizing the outcome (<70% redemption rate vs 70%-100% redemption rate) and treating redemption as an ordinal variable were run to provide measures of association that facilitate ease of interpretation. All analyses were conducted using SAS, version 9.4 (SAS Institute). A 2-sided *P* < .05 was considered statistically significant. The data were analyzed from May 22, 2024, to August 26, 2025.

## Results

A total of 188 368 infants and children (50.9% male; 46.9% aged 0-12 months at baseline; mean [SD], 1.5 years [1.2 years]) were included in the analysis. Redemption patterns varied significantly by age and participant category (Table 1); the group with the highest proportion of a total redemption rate of 70% or higher were partially breastfed infants (56.0%)—who receive some infant formula from WIC—and the group with the lowest rate were fully breastfed infants (45.5%)—who do not receive infant formula from WIC. In terms of race and ethnicity and language, 72.6% of other-language speaking non-Hispanic Asian participants and 69.6% of Spanish-speaking Hispanic participants had an overall redemption rate of 70% or higher, whereas only 22.2% of non-Hispanic Black participants had an overall redemption rate of 70% or higher.

**Table 1.** Characteristics of Infant and Child Participants, Caregivers, and Households by Categories of Interval-Scaled Total WIC Benefit Redemption Across Certification Periods^a^

Variable	No. (row %) of participants aged 0-3 y participating in WIC in Southern California, by redemption category							
	<10%	10% to <20%	20% to <30%	30% to <40%	40% to <50%	50% to <60%	60% to <70%	≥70%
No. of certification periods	19 089	14 205	18 849	24 698	31 307	37 011	41 977	179 076
Infant or child								
Participant category								
Formula-fed infant (n = 55 190)	1056 (1.9)	1281 (2.3)	2398 (4.3)	3870 (7.0)	5383 (9.8)	6823 (12.4)	7644 (13.9)	26 735 (48.4)
Partially breastfed infant (n = 32 070)	705 (2.2)	692 (2.2)	1156 (3.6)	1674 (5.2)	2567 (8.0)	3252 (10.1)	4080 (12.7)	17 944 (56.0)
Fully breastfed infant (n = 19 057)	892 (4.7)	813 (4.3)	1072 (5.6)	1482 (7.8)	1796 (9.4)	1989 (10.4)	2337 (12.3)	8676 (45.5)
Child aged 1 to <2 y (n = 87 534)	5664 (6.5)	4065 (4.6)	5000 (5.7)	6197 (7.1)	7419 (8.5)	8747 (10.0)	9639 (11.0)	40 803 (46.6)
Child aged 2 to <3 y (n = 87 720)	5677 (6.5)	3877 (4.4)	4838 (5.5)	5929 (6.8)	7335 (8.4)	8332 (9.5)	9323 (10.6)	42 409 (48.3)
Child aged 3 to <4 y (n = 84 641)	5095 (6.0)	3477 (4.1)	4385 (5.2)	5546 (6.6)	6807 (8.0)	7868 (9.3)	8954 (10.6)	42 509 (50.2)
Sex								
Male (n = 185 968)	9793 (5.3)	7160 (3.9)	9585 (5.2)	12 622 (6.8)	16 028 (8.6)	18 703 (10.1)	21 254 (11.4)	90 823 (48.8)
Female (n = 180 244)	9296 (5.1)	7045 (3.9)	9624 (5.3)	12 076 (7.0)	15 279 (8.5)	18 308 (10.1)	20 723 (11.5)	88 253 (48.9)
Race, ethnicity, and language^b^								
Non-Hispanic Asian, English speaking (n = 9602)	523 (5.4)	413 (4.3)	503 (5.2)	670 (7.0)	791 (8.2)	919 (9.6)	1022 (10.6)	4761 (49.6)
Non-Hispanic Asian, other language (n = 14 811)	396 (2.7)	229 (1.5)	354 (2.4)	495 (3.3)	638 (4.3)	866 (5.8)	1080 (7.3)	10 753 (72.6)
Non-Hispanic Black (n = 20 812)	2738 (13.2)	1964 (9.4)	2255 (10.8)	2502 (12.0)	2443 (11.7)	2250 (10.8)	2049 (9.8)	4611 (22.2)
Hispanic, English speaking (n = 208 482)	11 533 (5.5)	8895 (4.3)	12 128 (5.8)	16 378 (7.9)	20 786 (10.0)	24 410 (11.7)	26 822 (12.9)	87 530 (42.0)
Hispanic, Spanish speaking (n = 88 837)	1884 (2.1)	1349 (1.5)	1938 (2.2)	2730 (3.1)	4388 (4.9)	6222 (7.0)	8499 (9.6)	61 827 (69.6)
Non-Hispanic other (n = 12 771)	1042 (8.2)	734 (5.7)	894 (7.0)	1000 (7.8)	1199 (9.4)	1241 (9.7)	1299 (10.2)	5362 (42.0)
Non-Hispanic White (n = 10 897)	973 (8.9)	621 (5.7)	777 (7.1)	923 (8.5)	1062 (9.7)	1103 (10.1)	1206 (11.1)	4232 (38.8)
Caregiver or household								
≥1 mo Missing issuance in:								
Last 12 mo (n = 86 208)	5834 (6.8)	3686 (4.3)	4700 (5.5)	5876 (6.8)	7411 (8.6)	8348 (9.7)	9553 (11.1)	40 800 (47.3)
Last 6 mo (n = 37 627)	2985 (7.9)	1929 (5.1)	2382 (6.3)	2904 (7.7)	3427 (9.1)	3766 (10.0)	4172 (11.1)	16 062 (42.7)
Last 3 mo (n = 21 913)	2090 (9.5)	1191 (5.4)	1484 (6.8)	1740 (7.9)	2023 (9.2)	2198 (10.0)	2357 (10.8)	8830 (40.3)
Caregiver education								
Less than high school (n = 87 966)	3871 (4.4)	2785 (3.2)	3583 (4.1)	4884 (5.6)	6296 (7.2)	7818 (8.9)	9176 (10.4)	49 553 (56.3)
Completed high school (n = 161 563)	9043 (5.6)	6806 (4.2)	9059 (5.6)	11 712 (7.2)	14 691 (9.1)	17 154 (10.6)	18 978 (11.7)	74 120 (45.9)
More than high school (n = 116 683)	6175 (5.3)	4614 (4.0)	6207 (5.3)	8102 (6.9)	10 320 (8.8)	12 039 (10.3)	13 823 (11.8)	55 403 (47.5)
Medicaid participation (n = 311 339)	15 415 (5.0)	11 976 (3.8)	15 994 (5.1)	21 023 (6.8)	26 739 (8.6)	31 446 (10.1)	35 628 (11.4)	153 118 (49.2)
SNAP participation (n = 134 147)	7826 (5.8)	6183 (4.6)	8169 (6.1)	10 599 (7.9)	12 856 (9.6)	14 143 (10.5)	15 228 (11.4)	59 143 (44.1)
Household income, federal poverty level								
<50% (n = 99 963)	7257 (7.3)	5149 (5.2)	6333 (6.3)	7908 (7.9)	9490 (9.5)	10 521 (10.5)	11 209 (11.2)	42 096 (42.1)
50% to <100% (n = 136 156)	6156 (4.5)	4826 (3.5)	6521 (4.8)	8703 (6.4)	11 110 (8.2)	13 430 (9.9)	15 591 (11.5)	69 819 (51.3)
≥100% (n = 130 093)	5676 (4.4)	4230 (3.3)	5995 (4.6)	8087 (6.2)	10 707 (8.2)	13 060 (10.0)	15 177 (11.7)	67 161 (51.6)
Household size								
<4 People (n = 141 978)	10 359 (7.3)	7039 (5.0)	8881 (6.3)	11 105 (7.8)	13 240 (9.3)	14 892 (10.5)	16 266 (11.5)	60 196 (42.4)
4-5 People (n = 176 866)	7156 (4.0)	5800 (3.3)	8182 (4.6)	10 995 (6.2)	14 484 (8.2)	17 663 (10.0)	20 477 (11.6)	92 109 (52.1)
≥6 People (n = 47 368)	1574 (3.3)	1366 (2.9)	1786 (3.8)	2598 (5.5)	3583 (7.6)	4456 (9.4)	5234 (11.0)	26 771 (56.5)
Family members on WIC, No.								
1 (n = 150 152)	11 621 (7.7)	7092 (4.7)	8385 (5.6)	9834 (6.5)	11 928 (7.9)	13 818 (9.2)	15 595 (10.4)	71 879 (47.9)
2 (n = 144 040)	5772 (4.0)	4949 (3.4)	6961 (4.8)	9724 (6.8)	12 581 (8.7)	15 221 (10.6)	17 516 (12.2)	71 316 (49.5)
≥3 (n = 72 020)	1696 (2.4)	2164 (3.0)	3503 (4.9)	5140 (7.1)	6798 (9.4)	7972 (11.1)	8866 (12.3)	35 881 (49.8)
Mean (SD) No. of months of certification during:								
Cash-value benefit augment	7.2 (5.0)	7.3 (5.0)	7.5 (4.9)	7.6 (5.0)	7.5 (5.0)	7.4 (5.0)	7.3 (5.1)	7.0 (5.2)
Brand or package size flexibilities	4.9 (4.8)	4.8 (4.7)	4.5 (4.7)	4.5 (4.7)	4.5 (4.7)	4.6 (4.7)	4.6 (4.7)	4.8 (4.7)
Infant formula flexibilities	2.6 (3.8)	2.8 (3.9)	3.0 (4.0)	3.1 (4.0)	3.1 (4.1)	3.1 (4.1)	3.1 (4.1)	3.0 (4.0)

Abbreviations: SNAP, Supplemental Nutrition Assistance Program; WIC, Special Supplemental Nutrition Program for Women, Infants, and Children.

^a^ Certification periods included were those beginning between November 2019 and June 2022 (366 212 certification periods).

^b^ Grouped into a single variable (see Study Measures in Methods).

As shown in Table 1, 56.3% of caregivers with less than a high school education, 49.2% of Medicaid participants, 44.1% of SNAP participants, and 42.1% of households with incomes less than 50% of the FPL redeemed 70% or more of their benefits. In general, larger households and those with more family members in WIC had higher redemption rates than smaller households and households with fewer family members in WIC, respectively. The proportion of discontinued WIC participation decreased in a linear fashion as the total redemption of all foods increased; the WIC program discontinuation rate was 61.7% (11 768 discontinued participation of 19 089 certification periods) in the less than 10% redemption category, 42.0% (5961 discontinuations of 14 205 certification periods) in the 10% to less than 20% category, 33.8% (6366 discontinuations of 18 849 certification periods) in the 20% to less than 30% category, 27.3% (6747 discontinuations of 24 698 certification periods) in the 30% to less than 40% category, 22.8% (7146 discontinuations of 31 307 certification periods) in the 40% to less than 50% category, 19.0% (7043 discontinuations of 37 011 certification periods) in the 50% to less than 60% category, 16.5% (6917 discontinuations of 41 977 certification periods) in the 60% to less than 70% category, and 13.1% (23 521 discontinuations of 179 076 certification periods) in the 70% or greater category.

Infant formula was the WIC food category most redeemed, with 86.0% of households redeeming 70% or more of their issued infant formula benefits (Table 2). Fruits and vegetables were the second most redeemed, with 69.6% of households redeeming 70% or more of their issued fruit and vegetables benefits. Eggs (67.1% redeeming ≥70% of benefits) and whole milk (61.4% redeeming ≥70% of benefits) were also popular. The food category with the lowest uptake was infant meats, with 15.7% of households redeeming 70% or more of their issued infant meats benefit (Table 2).

**Table 2.** Distribution of Household Redemption of Category-Specific WIC Benefits^a^

Category	No. of certification periods	No. (row %)							
		<10% (Lowest)	10% to <20%	20% to <30%	30% to <40%	40% to <50%	50% to <60%	60% to <70%	≥70% (Highest)
FV	365 738	17 318 (4.7)	8708 (2.4)	10 409 (2.8)	12 330 (3.4)	15 374 (4.2)	20 247 (5.5)	26 846 (7.3)	254 506 (69.6)
Eggs	364 908	21 918 (6.0)	9726 (2.7)	11 320 (3.1)	12 683 (3.5)	13 356 (3.7)	25 685 (7.0)	25 445 (7.0)	244 775 (67.1)
Whole milk	176 981	21 106 (11.9)	4781 (2.7)	4337 (2.5)	9149 (5.2)	5129 (2.9)	9503 (5.4)	14 334 (8.1)	108 642 (61.4)
Cheese or tofu	365 031	30 236 (8.3)	13 747 (3.8)	16 076 (4.4)	16 817 (4.6)	16 663 (4.6)	30 351 (8.3)	28 080 (7.7)	213 061 (58.4)
100% FV juice	365 233	32 056 (8.8)	15 884 (4.3)	18 154 (5.0)	19 378 (5.3)	21 763 (6.0)	32 484 (8.9)	37 598 (10.3)	187 916 (51.5)
Reduced fat milk	335 907	44 849 (13.4)	18 049 (5.4)	17 613 (5.2)	19 434 (5.8)	18 688 (5.6)	23 528 (7.0)	26 920 (8.0)	166 826 (49.7)
Legumes	365 225	44 413 (12.2)	22 768 (6.2)	24 189 (6.6)	23 327 (6.4)	21 742 (6.0)	34 045 (9.3)	29 398 (8.0)	165 343 (45.3)
Canned fish^b^	45 164	11 159 (24.7)	1840 (4.1)	1966 (4.4)	2061 (4.6)	1809 (4.0)	3146 (7.0)	2791 (6.2)	20 392 (45.2)
Breakfast cereal	365 228	43 911 (12.0)	24 482 (6.7)	24 949 (6.8)	24 930 (6.8)	25 268 (6.9)	29 720 (8.1)	30 187 (8.3)	161 781 (44.3)
Bread or whole grain	355 763	54 039 (15.2)	20 889 (5.9)	22 761 (6.4)	21 499 (6.0)	19 465 (5.5)	33 341 (9.4)	26 497 (7.4)	157 272 (44.2)
Yogurt	364 782	58 312 (16.0)	24 724 (6.8)	25 351 (6.9)	23 228 (6.4)	21 249 (5.8)	34 930 (9.6)	28 494 (7.8)	148 494 (40.7)
Infant formula	140 789	4964 (3.5)	1052 (0.7)	1416 (1.0)	1545 (1.1)	1641 (1.2)	4299 (3.1)	4799 (3.4)	121 073 (86.0)
Therapeutic formula	7689	493 (6.4)	92 (1.2)	126 (1.6)	160 (2.1)	171 (2.2)	607 (7.9)	554 (7.2)	5486 (71.3)
Infant FV	151 096	25 202 (16.7)	10 611 (7.0)	9549 (6.3)	11 125 (7.4)	8700 (5.8)	12 226 (8.1)	12 250 (8.1)	61 433 (40.7)
Infant cereal	150 903	33 053 (21.9)	15 363 (10.2)	9582 (6.3)	12 528 (8.3)	5398 (3.6)	12 313 (8.2)	13 243 (8.8)	49 423 (32.8)
Infant meats	26 577	14 797 (55.7)	2347 (8.8)	1154 (4.3)	1341 (5.0)	669 (2.5)	1103 (4.2)	992 (3.7)	4174 (15.7)
Total redemption	366 212	19 089 (5.2)	14 205 (3.9)	18 849 (5.1)	24 698 (6.7)	31 307 (8.5)	37 011 (10.1)	41 977 (11.5)	179 076 (48.9)

Abbreviations: FV, fruits and vegetables; WIC, Special Supplemental Nutrition Program for Women, Infants, and Children.

^a^ Among households containing a WIC participant 0 to 3 years of age at certification in Southern California from November 2019 to June 2022 (366 212 certification periods).

^b^ Canned fish is a benefit provided only to exclusively breastfeeding postpartum women (3.8% of all WIC participants).

Overall, lower redemption (<70% compared with 70%-100%) of fruits and vegetables, infant formula, eggs, and whole milk were consistently associated with higher risk of program discontinuation, with the risk decreasing in a somewhat linear fashion as redemption rates increase (Table 3). For example, redeeming less than 10%, 10% to less than 20%, 20% to less than 30%, 30% to less than 40%, 40% to less than 50%, 50% to less than 60%, and 60% to less than 70% of fruit and vegetables issued benefits was associated with a risk of program discontinuation that was 2.08 (95% CI, 2.01-2.14), 1.77 (95% CI, 1.71-1.83), 1.73 (95% CI, 1.67-1.78), 1.54 (95% CI, 1.50-1.59), 1.48 (95% CI, 1.44-1.52), 1.37 (95% CI. 1.33-1.41), and 1.24 (95% CI, 1.21-1.27) times higher, respectively, compared with redeeming 70% to 100% of fruit and vegetables issued benefits (Table 3). Results were qualitatively similar when redemption was treated as dichotomous or ordinal; for example, redeeming less than 70% of fruit and vegetables issued benefits was associated with 35% higher risk of program discontinuation compared with redeeming 70% to 100% of the benefit, and each category decrease in redemption (ie, going from 70% to 60%) was associated with 10% higher risk of program discontinuation (eTable 2 in Supplement 1). Overall, the patterns observed for fruits and vegetables, eggs, whole milk, and infant formula were stronger in magnitude among participants younger than 2 years compared with participants 2 years or older (Figure; Table 4; eTable 3 in Supplement 1).

**Table 3.** Association of Household Category-Specific WIC Benefit Redemption With Risk of Discontinued WIC Participation^a^^,^^b^

Category	Interval-scaled category-specific benefit redemption, RR (95% CI)							
	<10%	10% to <20%	20% to <30%	30% to <40%	40% to <50%	50% to <60%	60% to <70%	70% to 100%
FV	2.08 (2.01-2.14)^c^	1.77 (1.71-1.83)^c^	1.73 (1.67-1.78)^c^	1.54 (1.50-1.59)^c^	1.48 (1.44-1.52)^c^	1.37 (1.33-1.41)^c^	1.24 (1.21-1.27)^c^	1.00 [Reference]
Eggs	1.64 (1.59-1.70)^c^	1.45 (1.40-1.50)^c^	1.36 (1.31-1.41)^c^	1.32 (1.28-1.37)^c^	1.26 (1.22-1.31)^c^	1.16 (1.13-1.19)^c^	1.14 (1.11-1.18)^c^	1.00 [Reference]
Whole milk	1.69 (1.63-1.76)^c^	1.36 (1.29-1.43)^c^	1.32 (1.25-1.39)^c^	1.21 (1.16-1.27)^c^	1.30 (1.24-1.37)^c^	1.19 (1.13-1.24)^c^	1.11 (1.07-1.16)^c^	1.00 [Reference]
Cheese or tofu	1.13 (1.09-1.17)^c^	1.02 (0.99-1.06)	1.00 (0.97-1.04)	1.02 (0.99-1.05)	0.97 (0.94-0.998)^c^	0.97 (0.94-0.99)^c^	1.02 (0.99-1.04)	1.00 [Reference]
100% FV juice	1.02 (0.99-1.05)	0.94 (0.91-0.98)^c^	0.95 (0.92-0.98)^c^	0.94 (0.91-0.97)^c^	0.93 (0.91-0.96)^c^	0.93 (0.90-0.95)^c^	0.92 (0.90-0.95)^c^	1.00 [Reference]
Reduced fat milk	1.08 (1.05-1.12)^c^	0.96 (0.93-0.99)^c^	0.99 (0.96-1.02)	0.98 (0.95-1.01)	1.00 (0.97-1.03)	1.00 (0.97-1.03)	0.97 (0.95-1.002)	1.00 [Reference]
Legumes	0.78 (0.75-0.80)^c^	0.73 (0.71-0.76)^c^	0.76 (0.74-0.79)^c^	0.79 (0.77-0.82)^c^	0.80 (0.78-0.83)^c^	0.83 (0.80-0.85)^c^	0.89 (0.87-0.92)^c^	1.00 [Reference]
Breakfast cereal	0.78 (0.75-0.80)^c^	0.75 (0.73-0.78)^c^	0.76 (0.73-0.78)^c^	0.78 (0.76-0.81)^c^	0.83 (0.80-0.85)^c^	0.85 (0.83-0.87)^c^	0.89 (0.87-0.92)^c^	1.00 [Reference]
Bread or whole grain	0.82 (0.79-0.84)^c^	0.77 (0.74-0.79)^c^	0.77 (0.75-0.80)^c^	0.82 (0.79-0.85)^c^	0.81 (0.79-0.84)^c^	0.86 (0.84-0.89)^c^	0.90 (0.88-0.93)^c^	1.00 [Reference]
Yogurt	0.70 (0.67-0.72)^c^	0.71 (0.69-0.74)^c^	0.74 (0.71-0.76)^c^	0.76 (0.74-0.78)^c^	0.77 (0.74-0.79)^c^	0.81 (0.79-0.84)^c^	0.90 (0.87-0.92)^c^	1.00 [Reference]
Infant formula	1.15 (1.10-1.20)^c^	1.30 (1.21-1.40)^c^	1.21 (1.12-1.30)^c^	1.26 (1.17-1.34)^c^	1.27 (1.18-1.36)^c^	1.16 (1.11-1.23)^c^	1.14 (1.09-1.20)^c^	1.00 [Reference]
Therapeutic formula	0.93 (0.77-1.12)	0.80 (0.57-1.13)	0.90 (0.64-1.25)	1.21 (0.94-1.55)	1.10 (0.85-1.44)	1.03 (0.88-1.22)	1.03 (0.86-1.24)	1.00 [Reference]
Infant FV	1.05 (1.01-1.09)^c^	0.90 (0.85-0.94)^c^	0.88 (0.83-0.93)^c^	0.99 (0.94-1.04)	0.88 (0.83-0.94)^c^	0.99 (0.94-1.04)	0.95 (0.90-1.01)	1.00 [Reference]
Infant cereal	0.88 (0.84-0.91)^c^	0.79 (0.75-0.83)^c^	0.78 (0.73-0.82)^c^	0.86 (0.82-0.91)^c^	0.86 (0.80-0.93)^c^	0.93 (0.88-0.98)^c^	0.89 (0.84-0.94)^c^	1.00 [Reference]
Infant meats	0.76 (0.67-0.86)^c^	0.71 (0.60-0.84)^c^	0.88 (0.73-1.06)	0.84 (0.68-1.02)	1.03 (0.82-1.29)	0.95 (0.77-1.17)	0.78 (0.61-0.999)^c^	1.00 [Reference]

Abbreviations: FV, fruits and vegetables; RR, risk ratio; WIC, Special Supplemental Nutrition Program for Women, Infants, and Children.

^a^ Among WIC participants 0 to 3 years of age at eligibility certification in Southern California from November 2019 to June 2022 (366 212 certification periods).

^b^ Associations are presented as RR (95% CI), and were determined in multivariable generalized estimating equation Poisson regression models for (dependent variable) discontinuation of WIC participation, regressed on (independent variables) sex, race and ethnicity language preference, and age category; interval-scaled category-specific benefit redemption at the household level and any months of missing issuance (yes or no); family representative educational attainment; and household Medicaid participation, SNAP participation, income category, size, the number of WIC participants, and the average total WIC food benefit redemption percentage (continuous) across the certification period; and the number of months during the certification period in which an augmented FV benefit was issued, the number of months in which brand or package size flexibility waivers were in place, and the number of months in which infant formula brand and package size flexibility waivers were in place. Models accommodated clustering of multiple certification periods for individual children and multiple children in families.

^c^ Indicates values that are statistically significant at *P* < .05.

**Figure.**
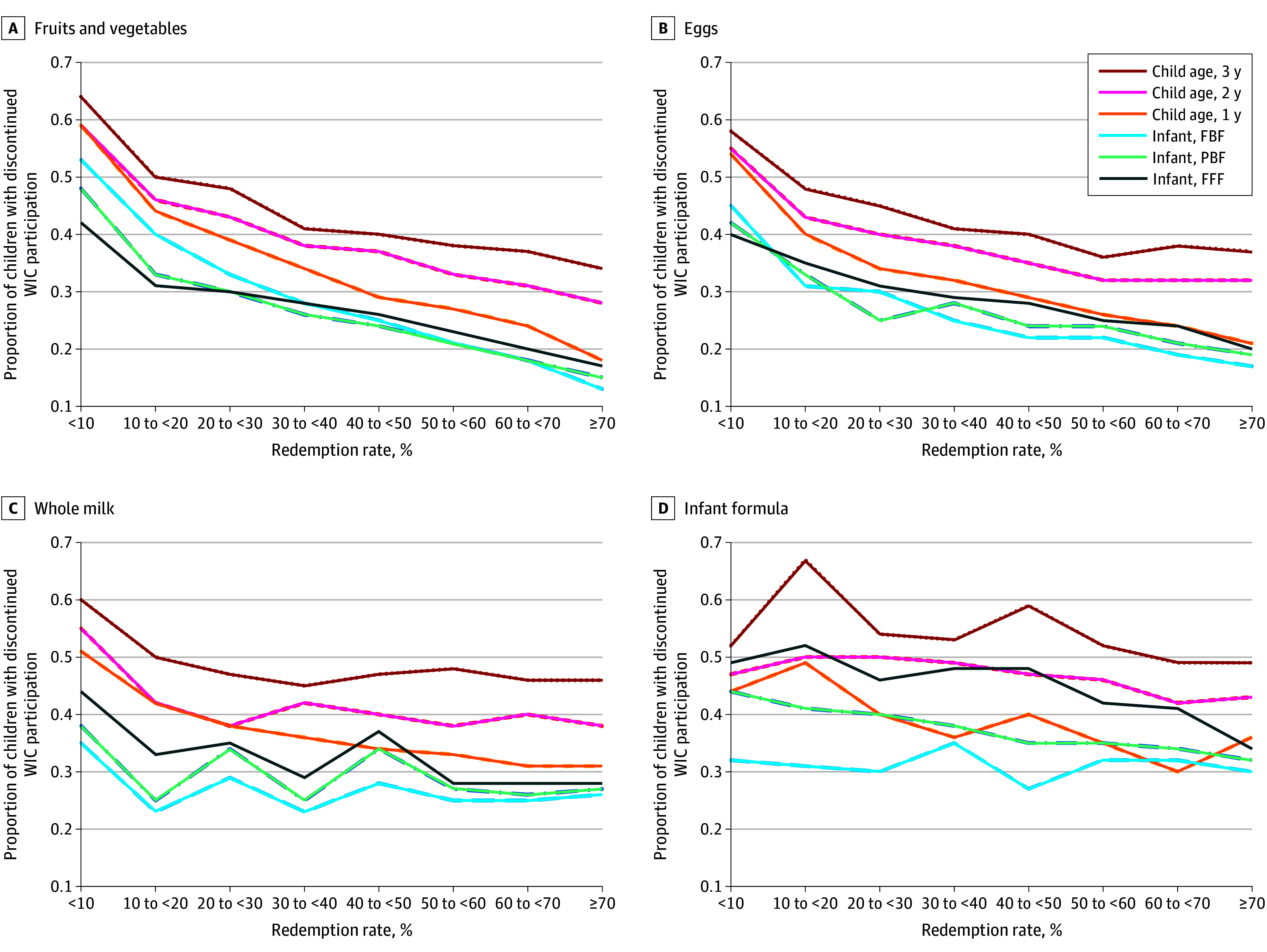
Proportion of Participants Who Discontinued Special Supplemental Nutrition Program for Women, Infants, and Children (WIC) by WIC Food and by Participant Category The proportion of participants with discontinued WIC participation by category-specific WIC benefit redemption and participant age or category are shown. This was estimated in multivariable generalized estimating equation linear regression models for (dependent variable) discontinuation of WIC participation, regressed on (independent variables) sex, race and ethnicity language preference, and age category; interval-scaled category-specific benefit redemption and any months of missing issuance (yes or no); family representative educational attainment; and household Medicaid participation, SNAP participation, income category, size, the number of WIC participants, and the average total WIC food benefit redemption percentage (continuous) across the certification period; the number of months during the certification period in which an augmented fruit and vegetable benefit was issued, the number of months in which brand or package size flexibility waivers were in place, and the number of months in which infant formula brand and package size flexibility waivers were in place; and a 2-way interaction of participant age and category with interval-scaled category-specific benefit redemption. Models accommodated clustering of multiple certification periods for individual children and multiple children in families. FBF indicates fully breastfed; FFF, fully formula fed; and PBF, partially breastfed.

**Table 4.** Association of Household Category-Specific WIC Benefit Redemption With Risk of Discontinued WIC Participation by Participant Category^a^^,^^b^

Food, participant category	Interval-scaled category-specific benefit redemption, RR (95% CI)								*P* value^c^
	<10%	10% to <20%	20% to <30%	30% to <40%	40% to <50%	50% to <60%	60% to <70%	70% to 100%	
Fruits and vegetables									
Formula-fed infant	2.76 (2.60-2.93)^d^	2.23 (2.06-2.43)^d^	2.23 (2.07-2.41)^d^	2.04 (1.90-2.20)^d^	1.93 (1.80-2.08)^d^	1.69 (1.57-1.81)^d^	1.44 (1.34-1.54)^d^	1.00 [Reference]	<.001
Partial BF infant	3.41 (3.15-3.69)^d^	2.73 (2.44-3.06)^d^	2.56 (2.29-2.86)^d^	2.24 (2.00-2.51)^d^	2.12 (1.91-2.35)^d^	1.88 (1.70-2.08)^d^	1.56 (1.41-1.72)^d^	1.00 [Reference]	
Fully BF infant	4.27 (3.90-4.68)^d^	3.75 (3.31-4.24)^d^	3.20 (2.81-3.65)^d^	2.82 (2.45-3.23)^d^	2.56 (2.25-2.92)^d^	2.16 (1.89-2.47)^d^	1.85 (1.62-2.10)^d^	1.00 [Reference]	
Child, age 1 y	3.39 (3.25-3.54)^d^	2.85 (2.69-3.01)^d^	2.64 (2.50-2.79)^d^	2.36 (2.23-2.50)^d^	2.10 (1.98-2.23)^d^	1.98 (1.88-2.10)^d^	1.68 (1.59-1.78)^d^	1.00 [Reference]	
Child, age 2 y	1.64 (1.57-1.70)^d^	1.40 (1.33-1.47)^d^	1.39 (1.32-1.46)^d^	1.24 (1.17-1.30)^d^	1.26 (1.20-1.33)^d^	1.13 (1.08-1.19)^d^	1.08 (1.03-1.13)^d^	1.00 [Reference]	
Child, age 3 y	1.37 (1.32-1.42)^d^	1.19 (1.13-1.25)^d^	1.21 (1.15-1.27)^d^	1.08 (1.03-1.14)^d^	1.08 (1.03-1.13)^d^	1.06 (1.02-1.11)^d^	1.04 (1.00-1.08)	1.00 [Reference]	
Eggs									
Formula-fed infant	2.23 (2.11-2.37)^d^	2.08 (1.91-2.26)^d^	1.93 (1.78-2.10)^d^	1.85 (1.70-2.00)^d^	1.81 (1.66-1.98)^d^	1.61 (1.50-1.72)^d^	1.56 (1.45-1.67)^d^	1.00 [Reference]	<.001
Partial BF infant	2.52 (2.33-2.72)^d^	2.23 (1.99-2.50)^d^	1.72 (1.52-1.95)^d^	1.99 (1.77-2.23)^d^	1.76 (1.55-2.00)^d^	1.79 (1.62-1.97)^d^	1.44 (1.29-1.60)^d^	1.00 [Reference]	
Fully BF infant	3.23 (2.95-3.53)^d^	2.58 (2.26-2.95)^d^	2.53 (2.21-2.90)^d^	2.25 (1.95-2.59)^d^	1.93 (1.64-2.27)^d^	1.92 (1.69-2.19)^d^	1.63 (1.42-1.87)^d^	1.00 [Reference]	
Child, age 1 y	2.67 (2.56-2.80)^d^	2.25 (2.12-2.38)^d^	2.01 (1.89-2.14)^d^	1.97 (1.86-2.09)^d^	1.79 (1.69-1.91)^d^	1.60 (1.51-1.69)^d^	1.45 (1.36-1.54)^d^	1.00 [Reference]	
Child, age 2 y	1.25 (1.20-1.30)^d^	1.10 (1.05-1.16)^d^	1.06 (1.00-1.11)^d^	1.05 (1.00-1.11)	1.02 (0.97-1.08)	0.95 (0.91-1.00)^d^	0.95 (0.91-1.00)^d^	1.00 [Reference]	
Child, age 3 y	1.04 (1.00-1.08)	0.95 (0.91-1.00)	0.96 (0.91-1.00)	0.91 (0.87-0.96)	0.93 (0.89-0.98)	0.85 (0.82-0.89)	0.94 (0.90-0.98)	1.00 [Reference]	
Whole milk									
Formula-fed infant	2.58 (2.42-2.76)^d^	1.75 (1.50-2.06)^d^	1.95 (1.63-2.33)^d^	1.42 (1.27-1.58)^d^	2.17 (1.83-2.58)^d^	1.27 (1.08-1.48)^d^	1.24 (1.10-1.38)^d^	1.00 [Reference]	<.001
Partial BF infant	2.81 (2.54-3.10)^d^	1.33 (0.99-1.80)	2.32 (1.67-3.23)^d^	1.24 (1.02-1.50)^d^	2.41 (1.74-3.36)^d^	1.19 (0.93-1.53)	1.22 (1.02-1.46)^d^	1.00 [Reference]	
Fully BF infant	2.81 (2.46-3.21)^d^	1.49 (1.06-2.10)^d^	2.14 (1.48-3.10)^d^	1.23 (0.97-1.57)	2.04 (1.32-3.15)^d^	1.32 (0.97-1.79)	1.31 (1.04-1.64)^d^	1.00 [Reference]	
Child, age 1 y	1.63 (1.55-1.71)^d^	1.51 (1.42-1.60)^d^	1.42 (1.34-1.52)^d^	1.39 (1.31-1.48)^d^	1.35 (1.26-1.44)^d^	1.29 (1.22-1.37)^d^	1.19 (1.12-1.26)^d^	1.00 [Reference]	
Child, age 2 y	1.15 (1.06-1.24)^d^	0.91 (0.78-1.07)	0.83 (0.70-0.99)	1.02 (0.89-1.17)	0.98 (0.83-1.16)	0.93 (0.81-1.08)	1.04 (0.92-1.18)	1.00 [Reference]	
Child, age 3 y	0.85 (0.80-0.91)^d^	0.76 (0.69-0.85)^d^	0.74 (0.66-0.84)^d^	0.75 (0.67-0.83)^d^	0.83 (0.74-0.94)^d^	0.88 (0.81-0.97)^d^	0.88 (0.80-0.96)^d^	1.00 [Reference]	
Cheese or tofu									
Formula-fed infant	1.54 (1.45-1.63)^d^	1.52 (1.40-1.64)^d^	1.51 (1.40-1.63)^d^	1.46 (1.35-1.58)^d^	1.34 (1.22-1.46)^d^	1.41 (1.31-1.50)^d^	1.32 (1.23-1.42)^d^	1.00 [Reference]	<.001
Partial BF infant	1.75 (1.62-1.89)^d^	1.29 (1.15-1.45)^d^	1.39 (1.24-1.56)^d^	1.44 (1.29-1.62)^d^	1.33 (1.17-1.52)^d^	1.37 (1.24-1.51)^d^	1.23 (1.11-1.37)^d^	1.00 [Reference]	
Fully BF infant	2.09 (1.90-2.30)^d^	1.61 (1.40-1.84)^d^	1.55 (1.35-1.77)^d^	1.41 (1.23-1.63)^d^	1.31 (1.12-1.54)^d^	1.35 (1.18-1.55)^d^	1.32 (1.14-1.52)^d^	1.00 [Reference]	
Child, age 1 y	1.83 (1.75-1.92)^d^	1.52 (1.43-1.61)^d^	1.43 (1.35-1.52)^d^	1.46 (1.37-1.55)^d^	1.29 (1.21-1.37)^d^	1.31 (1.24-1.38)^d^	1.26 (1.19-1.35)^d^	1.00 [Reference]	
Child, age 2 y	0.84 (0.80-0.87)^d^	0.81 (0.77-0.85)^d^	0.79 (0.75-0.83)^d^	0.82 (0.78-0.86)^d^	0.83 (0.79-0.87)^d^	0.79 (0.76-0.83)^d^	0.89 (0.85-0.93)^d^	1.00 [Reference]	
Child, age 3 y	0.71 (0.68-0.74)^d^	0.70 (0.66-0.73)^d^	0.71 (0.68-0.74)^d^	0.74 (0.70-0.77)^d^	0.73 (0.69-0.76)^d^	0.74 (0.71-0.77)^d^	0.85 (0.81-0.89)^d^	1.00 [Reference]	
100% Juice									
Formula-fed infant	1.62 (1.51-1.72)^d^	1.45 (1.33-1.57)^d^	1.45 (1.34-1.57)^d^	1.51 (1.40-1.63)^d^	1.48 (1.38-1.60)^d^	1.35 (1.25-1.45)^d^	1.22 (1.14-1.31)^d^	1.00 [Reference]	<.001
Partial BF infant	1.68 (1.55-1.83)^d^	1.49 (1.33-1.66)^d^	1.50 (1.34-1.67)^d^	1.35 (1.20-1.51)^d^	1.46 (1.32-1.62)^d^	1.36 (1.23-1.51)^d^	1.22 (1.11-1.35)^d^	1.00 [Reference]	
Fully BF infant	1.96 (1.78-2.16)^d^	1.66 (1.46-1.90)^d^	1.62 (1.42-1.86)^d^	1.38 (1.19-1.60)^d^	1.46 (1.27-1.68)^d^	1.35 (1.17-1.56)^d^	1.26 (1.09-1.45)^d^	1.00 [Reference]	
Child, age 1 y	1.66 (1.58-1.74)^d^	1.39 (1.31-1.47)^d^	1.41 (1.33-1.49)^d^	1.30 (1.22-1.38)^d^	1.25 (1.18-1.34)^d^	1.30 (1.23-1.37)^d^	1.15 (1.08-1.22)^d^	1.00 [Reference]	
Child, age 2 y	0.78 (0.75-0.81)^d^	0.75 (0.71-0.78)^d^	0.75 (0.72-0.79)^d^	0.78 (0.74-0.82)^d^	0.76 (0.73-0.80)^d^	0.78 (0.75-0.82)^d^	0.84 (0.80-0.88)^d^	1.00 [Reference]	
Child, age 3 y	0.66 (0.63-0.68)^d^	0.68 (0.65-0.71)^d^	0.69 (0.66-0.72)^d^	0.69 (0.66-0.72)^d^	0.70 (0.67-0.73)^d^	0.72 (0.69-0.75)^d^	0.80 (0.77-0.84)^d^	1.00 [Reference]	
Reduced fat milk									
Formula-fed infant	1.50 (1.42-1.59)^d^	1.44 (1.34-1.56)^d^	1.38 (1.28-1.50)^d^	1.44 (1.33-1.55)^d^	1.56 (1.44-1.69)^d^	1.30 (1.19-1.41)^d^	1.33 (1.23-1.45)^d^	1.00 [Reference]	<.001
Partial BF infant	1.65 (1.53-1.79)^d^	1.44 (1.29-1.61)^d^	1.57 (1.42-1.75)^d^	1.60 (1.43-1.78)^d^	1.46 (1.30-1.64)^d^	1.43 (1.28-1.60)^d^	1.31 (1.16-1.47)^d^	1.00 [Reference]	
Fully BF infant	1.97 (1.77-2.19)^d^	1.57 (1.37-1.80)^d^	1.47 (1.27-1.71)^d^	1.62 (1.39-1.88)^d^	1.34 (1.14-1.59)^d^	1.37 (1.15-1.63)^d^	1.27 (1.07-1.51)^d^	1.00 [Reference]	
Child, age 1 y	1.82 (1.73-1.92)^d^	1.45 (1.34-1.56)^d^	1.40 (1.29-1.52)^d^	1.34 (1.23-1.46)^d^	1.51 (1.38-1.65)^d^	1.42 (1.30-1.54)^d^	1.28 (1.17-1.39)^d^	1.00 [Reference]	
Child, age 2 y	0.83 (0.80-0.86)^d^	0.81 (0.77-0.85)^d^	0.81 (0.77-0.85)^d^	0.83 (0.79-0.87)^d^	0.84 (0.80-0.88)^d^	0.87 (0.83-0.91)^d^	0.87 (0.82-0.91)^d^	1.00 [Reference]	
Child, age 3 y	0.69 (0.66-0.71)^d^	0.66 (0.63-0.69)^d^	0.75 (0.71-0.78)^d^	0.73 (0.70-0.76)^d^	0.75 (0.71-0.78)^d^	0.80 (0.77-0.84)^d^	0.81 (0.78-0.85)^d^	1.00 [Reference]	
Legumes									
Formula-fed infant	1.19 (1.12-1.26)^d^	1.09 (1.01-1.18)^d^	1.15 (1.07-1.24)^d^	1.13 (1.05-1.23)^d^	1.12 (1.02-1.23)^d^	1.15 (1.07-1.24)^d^	1.12 (1.03-1.22)^d^	1.00 [Reference]	<.001
Partial BF infant	1.23 (1.14-1.34)^d^	1.08 (0.97-1.20)	1.14 (1.02-1.26)^d^	1.09 (0.97-1.22)	1.04 (0.91-1.18)	1.26 (1.14-1.39)^d^	1.13 (1.01-1.27)^d^	1.00 [Reference]	
Fully BF infant	1.45 (1.30-1.61)^d^	1.16 (1.01-1.32)^d^	1.21 (1.06-1.39)^d^	1.20 (1.04-1.40)^d^	1.12 (0.95-1.32)^d^	1.22 (1.05-1.42)^d^	1.19 (1.01-1.41)^d^	1.00 [Reference]	
Child, age 1 y	1.24 (1.17-1.30)^d^	1.04 (0.98-1.10)	1.04 (0.98-1.10)	1.06 (0.99-1.12)	1.04 (0.97-1.11)	1.04 (0.98-1.11)	1.02 (0.94-1.09)	1.00 [Reference]	
Child, age 2 y	0.56 (0.53-0.58)^d^	0.58 (0.55-0.60)^d^	0.59 (0.56-0.61)^d^	0.65 (0.62-0.68)^d^	0.68 (0.65-0.72)^d^	0.69 (0.66-0.73)^d^	0.77 (0.73-0.81)^d^	1.00 [Reference]	
Child, age 3 y	0.48 (0.46-0.50)^d^	0.50 (0.48-0.53)^d^	0.56 (0.53-0.58)^d^	0.59 (0.56-0.62)^d^	0.62 (0.59-0.65)^d^	0.65 (0.62-0.68)^d^	0.80 (0.76-0.83)^d^	1.00 [Reference]	
BF cereal									
Formula-fed infant	1.19 (1.12-1.26)^d^	1.12 (1.04-1.21)^d^	1.14 (1.06-1.23)^d^	1.11 (1.03-1.20)^d^	1.21 (1.11-1.31)^d^	1.11 (1.03-1.20)^d^	1.15 (1.06-1.24)^d^	1.00 [Reference]	<.001
Partial BF infant	1.17 (1.08-1.27)^d^	1.11 (1.00-1.23)^d^	1.08 (0.97-1.20)	1.11 (1.00-1.24)^d^	1.06 (0.94-1.19)	1.15 (1.04-1.28)^d^	1.05 (0.94-1.18)	1.00 [Reference]	
Fully BF infant	1.45 (1.31-1.61)^d^	1.24 (1.09-1.41)^d^	1.05 (0.91-1.21)	1.20 (1.04-1.38)^d^	1.31 (1.13-1.52)^d^	1.16 (1.00-1.36)^d^	1.22 (1.05-1.43)^d^	1.00 [Reference]	
Child, age 1 y	1.24 (1.18-1.30)^d^	1.06 (1.00-1.13)	1.05 (0.99-1.12)	1.03 (0.96-1.09)	1.06 (0.99-1.13)	1.08 (1.02-1.16)^d^	1.07 (1.00-1.15)	1.00 [Reference]	
Child, age 2 y	0.56 (0.54-0.59)^d^	0.59 (0.56-0.62)^d^	0.61 (0.58-0.64)^d^	0.63 (0.60-0.67)^d^	0.67 (0.64-0.70)^d^	0.73 (0.70-0.77)^d^	0.78 (0.74-0.82)^d^	1.00 [Reference]	
Child, age 3 y	0.48 (0.46-0.50)^d^	0.51 (0.49-0.54)^d^	0.53 (0.51-0.56)^d^	0.59 (0.56-0.62)^d^	0.65 (0.62-0.68)^d^	0.67 (0.65-0.70)^d^	0.76 (0.73-0.80)^d^	1.00 [Reference]	
Bread or whole grains									
Formula-fed infant	1.32 (1.25-1.40)^d^	1.21 (1.10-1.34)^d^	1.10 (0.99-1.22)^d^	1.26 (1.14-1.39)^d^	1.33 (1.18-1.49)^d^	1.10 (1.02-1.20)^d^	1.07 (0.95-1.20)	1.00 [Reference]	<.001
Partial BF infant	1.33 (1.23-1.43)^d^	1.13 (0.99-1.29)	1.04 (0.91-1.18)	1.17 (1.03-1.32)	1.14 (0.99-1.33)	1.20 (1.09-1.33)	1.10 (0.97-1.25)	1.00 [Reference]	
Fully BF infant	1.52 (1.38-1.67)^d^	1.15 (1.00-1.32)	1.25 (1.08-1.43)^d^	1.20 (1.04-1.38)^d^	1.16 (0.99-1.36)	1.11 (0.96-1.29)	1.18 (1.01-1.36)^d^	1.00 [Reference]	
Child, age 1 y	1.20 (1.14-1.27)^d^	1.04 (0.98-1.11)	1.06 (0.99-1.12)	1.04 (0.98-1.12)	1.02 (0.95-1.10)	1.12 (1.05-1.20)^d^	1.02 (0.95-1.10)	1.00 [Reference]	
Child, age 2 y	0.56 (0.53-0.58)^d^	0.58 (0.55-0.61)^d^	0.60 (0.57-0.63)^d^	0.64 (0.61-0.68)^d^	0.67 (0.64-0.71)^d^	0.73 (0.70-0.77)^d^	0.82 (0.78-0.87)^d^	1.00 [Reference]	
Child, age 3 y	0.48 (0.46-0.50)^d^	0.52 (0.50-0.55)^d^	0.55 (0.52-0.57)^d^	0.61 (0.59-0.64)^d^	0.61 (0.58-0.64)^d^	0.68 (0.65-0.71)^d^	0.77 (0.74-0.81)^d^	1.00 [Reference]	
Yogurt									
Formula-fed infant	1.08 (1.02-1.14)^d^	1.09 (1.01-1.18)^d^	1.09 (1.01-1.17)^d^	1.05 (0.97-1.14)	1.05 (0.95-1.15)	1.08 (1.00-1.17)	1.16 (1.07-1.26)^d^	1.00 [Reference]	<.001
Partial BF infant	1.13 (1.04-1.22)^d^	0.97 (0.87-1.08)	1.06 (0.95-1.18)	0.97 (0.86-1.09)	1.00 (0.88-1.14)	1.10 (0.99-1.22)	1.00 (0.89-1.12)	1.00 [Reference]	
Fully BF infant	1.22 (1.10-1.35)^d^	1.05 (0.92-1.19)	1.06 (0.92-1.22)	1.06 (0.92-1.23)	1.00 (0.85-1.18)	1.03 (0.89-1.20)	1.12 (0.96-1.31)	1.00 [Reference]	
Child, age 1 y	1.04 (0.99-1.10)	0.94 (0.88-1.00)	0.96 (0.90-1.02)	0.95 (0.89-1.02)	0.94 (0.87-1.01)	1.01 (0.94-1.08)	1.02 (0.94-1.10)	1.00 [Reference]	
Child, age 2 y	0.53 (0.51-0.55)^d^	0.59 (0.56-0.62)^d^	0.62 (0.59-0.65)^d^	0.66 (0.63-0.69)^d^	0.68 (0.64-0.72)^d^	0.71 (0.68-0.75)^d^	0.81 (0.77-0.86)^d^	1.00 [Reference]	
Child, age 3 y	0.45 (0.43-0.47)^d^	0.52 (0.50-0.54)^d^	0.54 (0.52-0.57)^d^	0.60 (0.57-0.63)^d^	0.62 (0.59-0.65)^d^	0.67 (0.64-0.70)^d^	0.79 (0.76-0.83)^d^	1.00 [Reference]	
Infant formula									
Formula-fed infant	1.32 (1.23-1.42)^d^	1.57 (1.42-1.73)^d^	1.41 (1.27-1.57)^d^	1.55 (1.40-1.71)^d^	1.63 (1.48-1.79)^d^	1.47 (1.36-1.60)^d^	1.48 (1.38-1.60)^d^	1.00 [Reference]	<.001
Partial BF infant	1.40 (1.29-1.51)^d^	1.47 (1.29-1.67)^d^	1.50 (1.33-1.69)^d^	1.48 (1.30-1.68)^d^	1.35 (1.17-1.54)^d^	1.39 (1.25-1.54)^d^	1.32 (1.19-1.46)^d^	1.00 [Reference]	
Fully BF infant	1.34 (1.17-1.54)^d^	1.30 (0.96-1.77)^d^	1.35 (1.02-1.78)^d^	1.67 (1.29-2.15)^d^	1.08 (0.74-1.58)	1.50 (1.20-1.89)^d^	1.40 (1.14-1.72)^d^	1.00 [Reference]	
Child, age 1 y	1.28 (1.18-1.38)^d^	1.36 (1.08-1.71)^d^	1.12 (0.87-1.44)	0.97 (0.73-1.29)	1.15 (0.90-1.47)	0.94 (0.80-1.10)	0.72 (0.57-0.91)	1.00 [Reference]	
Child, age 2 y	0.81 (0.72-0.91)^d^	0.88 (0.68-1.14)	0.97 (0.79-1.19)	1.00 (0.83-1.19)	0.90 (0.74-1.10)	0.93 (0.82-1.06)	0.87 (0.75-1.00)	1.00 [Reference]	
Child, age 3 y	0.75 (0.66-0.85)^d^	0.97 (0.80-1.17)	0.82 (0.68-0.98)^d^	0.87 (0.73-1.04)	1.00 (0.84-1.19)	0.91 (0.81-1.03)	0.88 (0.77-1.00)	1.00 [Reference]	
Therapeutic formula									
Formula-fed infant	0.97 (0.76-1.24)	0.54 (0.26-1.15)	1.07 (0.60-1.92)	1.16 (0.75-1.80)	0.98 (0.68-1.40)	1.03 (0.81-1.31)	0.99 (0.74-1.33)	1.00 [Reference]	.46
Partial BF infant	1.26 (0.87-1.83)	0.84 (0.36-1.96)	1.56 (1.00-2.41)	0.91 (0.44-1.87)	1.40 (0.91-2.18)	1.04 (0.74-1.45)	1.48 (1.08-2.04)^d^	1.00 [Reference]	
Fully BF infant	1.19 (0.59-2.41)	4.07 (0.73-22.58)	0.92 (0.11-7.95)	0.66 (0.17-2.52)	1.24 (0.62-2.49)	1.15 (0.66-2.00)	1.15 (0.60-2.22)	1.00 [Reference]	
Child, age 1 y	0.95 (0.63-1.43)	1.00 (0.56-1.76)	1.40 (0.79-2.49)	1.37 (0.86-2.17)	1.38 (0.54-3.52)	1.28 (0.82-2.00)	1.03 (0.65-1.64)	1.00 [Reference]	
Child, age 2 y	0.81 (0.52-1.27)	0.97 (0.38-2.50)	0.34 (0.07-1.64)	1.13 (0.47-2.71)	0.96 (0.38-2.44)	0.76 (0.42-1.37)	0.58 (0.30-1.14)	1.00 [Reference]	
Child-age 3 y	0.57 (0.29-1.12)	0.44 (0.18-1.09)	0.21 (0.06-0.81)^d^	1.64 (0.97-2.75)	1.08 (0.36-3.27)	1.03 (0.68-1.56)	1.20 (0.72-2.01)	1.00 [Reference]	
Infant fruits and vegetables									
Formula-fed infant	1.42 (1.33-1.52)^d^	1.16 (1.07-1.25)^d^	1.10 (1.01-1.20)^d^	1.25 (1.16-1.35)^d^	1.00 (0.91-1.11)	1.16 (1.07-1.26)^d^	1.11 (1.02-1.22)^d^	1.00 [Reference]	<.001
Partial BF infant	1.69 (1.55-1.83)^d^	1.26 (1.13-1.41)^d^	1.11 (0.98-1.25)	1.28 (1.14-1.43)^d^	1.10 (0.96-1.25)	1.22 (1.08-1.37)^d^	1.14 (1.01-1.29)^d^	1.00 [Reference]	
Fully BF infant	1.61 (1.44-1.81)^d^	1.12 (0.97-1.31)	1.14 (0.97-1.34)	1.16 (0.99-1.37)	1.01 (0.84-1.23)	1.23 (1.03-1.47)^d^	1.11 (0.91-1.35)	1.00 [Reference]	
Child, age 1 y	0.99 (0.92-1.05)^d^	0.80 (0.67-0.94)^d^	0.79 (0.66-0.95)^d^	0.84 (0.71-0.98)^d^	0.83 (0.68-1.03)	0.82 (0.69-0.97)^d^	0.67 (0.54-0.82)^d^	1.00 [Reference]	
Child, age 2 y	0.75 (0.70-0.82)^d^	0.70 (0.62-0.79)^d^	0.78 (0.69-0.87)^d^	0.81 (0.72-0.91)^d^	0.83 (0.72-0.95)^d^	0.98 (0.88-1.09)	0.92 (0.81-1.04)	1.00 [Reference]	
Child, age 3 y	0.68 (0.63-0.73)^d^	0.67 (0.60-0.74)^d^	0.67 (0.60-0.74)^d^	0.83 (0.75-0.92)^d^	0.79 (0.70-0.90)^d^	0.79 (0.71-0.88)^d^	0.86 (0.77-0.96)^d^	1.00 [Reference]	
Infant cereal									
Formula-fed infant	1.08 (1.01-1.16)^d^	0.96 (0.89-1.04)	0.89 (0.81-0.98)^d^	1.03 (0.95-1.12)	0.93 (0.82-1.05)	1.00 (0.91-1.10)	1.00 (0.91-1.09)	1.00 [Reference]	<.001
Partial BF infant	1.31 (1.20-1.44)^d^	1.04 (0.93-1.15)	0.92 (0.81-1.05)	1.15 (1.02-1.29)^d^	1.05 (0.89-1.23)	1.12 (0.99-1.27)	1.09 (0.96-1.23)	1.00 [Reference]	
Fully BF infant	1.25 (1.11-1.41)^d^	0.85 (0.74-0.99)^d^	0.87 (0.73-1.04)	0.98 (0.83-1.16)	1.01 (0.82-1.26)	1.12 (0.94-1.34)	0.87 (0.71-1.07)	1.00 [Reference]	
Child, age 1 y	0.89 (0.83-0.95)^d^	0.66 (0.53-0.81)^d^	0.75 (0.60-0.95)^d^	0.71 (0.59-0.85)^d^	0.87 (0.60-1.25)	0.70 (0.56-0.88)^d^	0.72 (0.61-0.86)^d^	1.00 [Reference]	
Child, age 2 y	0.68 (0.63-0.74)^d^	0.65 (0.58-0.73)^d^	0.75 (0.66-0.85)^d^	0.76 (0.67-0.86)^d^	0.79 (0.66-0.93)^d^	0.90 (0.80-1.01)	0.83 (0.72-0.94)^d^	1.00 [Reference]	
Child, age 3 y	0.60 (0.56-0.64)^d^	0.63 (0.57-0.70)^d^	0.62 (0.56-0.70)^d^	0.68 (0.61-0.76)^d^	0.77 (0.67-0.88)^d^	0.84 (0.76-0.94)^d^	0.86 (0.77-0.95)^d^	1.00 [Reference]	

Abbreviations: BF, breastfed; RR, risk ratio; SNAP, Supplemental Nutrition Assistance Program; WIC, Special Supplemental Nutrition Program for Women, Infants, and Children.

^a^ Among WIC participants 0 to 3 years of age at eligibility certification in Southern California from November 2019 to June 2022 (366 212 certification periods).

^b^ Associations are presented as RR (95% CI) and were determined in multivariable generalized estimating equation Poisson regression models for (dependent variable) discontinuation of WIC participation, regressed on (independent variables) sex, race and ethnicity language preference, and age category; interval-scaled category-specific benefit redemption at the household level and any months of missing issuance (yes or no); family representative educational attainment; and household Medicaid participation, SNAP participation, income category, size, the number of WIC participants, and the average total WIC food benefit redemption percentage (continuous) across the certification period; the number of months during the certification period in which an augmented fruit and vegetable benefit was issued, the number of months in which brand or package size flexibility waivers were in place, and the number of months in which infant formula brand and package size flexibility waivers were in place; and a 2-way interaction of participant age and category with interval-scaled category-specific benefit redemption. Models accommodated clustering of multiple certification periods for individual children and multiple children in families.

^c^ *P* value is for the type 3 test of the interaction between interval-scaled category-specific benefit redemption and participant category.

^d^ Statistically significant at *P* < .05.

Associations with a lower risk of program discontinuation were observed for lower redemption of breakfast cereal (risk ratios [RRs] between 0.75 [95% CI, 0.73-0.78] and 0.89 [95% CI, 0.87-0.92]), legumes (RRs between 0.73 [95% CI, 0.71-0.76] and 0.89 [95% CI, 0.87-0.92]), infant cereal (RRs between 0.78 [95% CI, 0.73-0.82] and 0.93 [95% CI, 0.88-0.98]), bread or whole grain (RRs between 0.77 [95% CI, 0.74-0.79] and 0.90 [95% CI, 0.87-0.93]), yogurt (RRs between 0.70 [95% CI, 0.67-0.72] and 0.90 [95% CI, 0.87-0.92]), and 100% fruit or vegetable juice (RRs between 0.92 [95% CI, 0.90-0.95] and 0.95 [95% CI, 0.92-0.98]) (Table 3). However, on stratification by age or category, these observed associations were present among participants 2 years or older only (Table 4; eTable 3 in Supplement 1). It is important to note that these results include adjustment by the total benefit redemption percentage; in models not adjusted for the total benefit redemption percentage, lower redemption of every WIC food was associated with increased risk of program discontinuation (eTable 4 in Supplement 1).

## Discussion

Among 188 368 WIC participants aged 0 to 3 years in Southern California, we found that fruits and vegetables and infant formula are the most redeemed WIC foods, which aligns with previous research in different states.^9,10,12,20^ We also identified participant characteristics associated with lower WIC food redemption rates; in particular, non-Hispanic Black children had lower redemption rates, a finding that has been reported previously^12,21^ and that merits further attention. We also found that redemption patterns of specific WIC foods were associated with risk of program discontinuation. As hypothesized, lower redemption (<70%) of infant formula, fruits and vegetables, eggs, and whole milk—the most highly redeemed WIC food categories—was associated with increased risk of program discontinuation, with the highest risk among those redeeming less than 10% (vs 70%-100%) of these food benefits. This finding was particularly true for participants younger than 2 years. The findings of smaller magnitude associations for participants 2 years or older suggest that, for households with older children, WIC program services other than WIC foods may contribute to continued participation. This aligns with prior national findings reporting that a higher perceived value of WIC nutrition education was associated with longer WIC participation.^2^ An important note is that models not adjusted for the total benefit redemption percentage show that lower redemption of every WIC food was associated with increased risk of program discontinuation. WIC food benefit redemption is correlated across categories (ie, those who redeem more of one benefit category are more likely to redeem more of every other benefit category), so our main study findings (adjusted for the total redemption percentage) show the independent association between redemption of each food and continued participation. Our results expand on previous findings that lower overall redemption of WIC foods was associated with risk of program discontinuation.^6^

Previous research identified the reasons why WIC families discontinue WIC participation while still eligible. Data collected as part of the National Health and Nutrition Examination Survey in 2019 to 2020 indicate that the most common reasons for no longer participating in WIC while remaining eligible were time constraints to attend a WIC clinic (20.4%) and receipt of benefits from other programs, including SNAP and food banks (12.7%).^22^ Nine percent of the sample also indicated issues (unspecified) with WIC foods and 7.6% not using the benefits.^22^ Qualitative studies in Illinois^23^ and Rhode Island^24^ identified barriers associated with WIC foods at the WIC vendor level, including incorrect labeling, inconsistency of WIC-approved foods, and confusion about sizes and types of WIC foods allowed per WIC food category; the latter in particular seemed to influence the level of benefit redemption. Participants also reported discontent over restrictive food choices (in particular compared with SNAP) and a lower perceived value of the child food package compared with the formula feeding infant packages.^23^ It is important to note that, unlike SNAP—which does not restrict the type of foods and beverages their participants can purchase with SNAP benefits—WIC provides prescribed healthy food packages tailored to each participant type, and participants receiving WIC benefits are required to engage in nutrition education.

Insufficient benefits for fruits and vegetables have also been reported as a reason for early WIC program discontinuation.^25^ However, that was before increases to fruit and vegetable benefits were introduced in 2021. This benefit increase has improved program satisfaction and fruit and vegetable redemption^13,14,15,16,17^ and has been made permanent in the new 2024 WIC food package, to be fully implemented by April 2026.^26^ A widely cited reason for early program discontinuation is feelings of embarrassment related to choosing foods that were later identified as not eligible during checkout.^23,24,27,28^ However, technological advances, including the introduction of a WIC Shopper app, have facilitated identification of WIC-allowed foods and reduced embarrassment, positively influencing redemption.^24,25,29^ Stigma-related issues may be further improved with the implementation of WIC online shopping opportunities.^30,31^

### Strengths and Limitations

Strengths of this study include the availability of rich redemption data from a very large WIC program, allowing us to explore how redemption patterns of specific WIC foods affected program discontinuation, and how this association varied by participant age and type. Further, our analysis was adjusted for average benefit redemption, allowing us to identify whether lower redemption of specific WIC food benefit categories was associated with risk of discontinued participation independently of overall benefit redemption.

This study also has some limitations, including the observational study design, which precludes causal inference. Also, the study dates overlap with the COVID-19 pandemic, a period characterized by supply chain issues leading to food shortages. WIC responded to COVID-19 with brand and package flexibilities; however, food shortages and changes to in-person shopping behaviors may have still affected redemption patterns. This study is based in Southern California, which may limit the generalizability of the findings to families that live in less urban areas, with access to fewer WIC-approved vendors, or with different racial and ethnic composition.

## Conclusions

This cohort study suggests that fruits and vegetables, eggs, infant formula, and whole milk are WIC participants’ most highly valued food benefits and that redemption patterns of these foods could assist in identifying children at risk for program discontinuation. Identifying children at risk of WIC discontinuation is crucial for WIC retention efforts, which could be realized via targeted text messages to families with low or no redemption and/or tailored nutrition education addressing how to incorporate WIC foods into children’s diets for families with low redemption. To maximize the positive outcomes of longer WIC participation—which include higher household food security and healthier dietary patterns,^2,3,4,5^ both clinically relevant given the associations of food insecurity with poor diet quality and risk of chronic disease^32,33^—future research investigating ways to address the reasons why WIC participants do not fully redeem their benefits is needed. Further, future efforts focusing on designing enhanced nutrition education tailored to WIC participants at risk of program discontinuation, focused on how to take full advantage of WIC food benefits, may have a substantial positive nutritional impact.

## Notes

Supplement 1.
eMethods.
**eFigure 1.** Flow chart for inclusion of Special Supplemental Nutrition Program for Women, Infants, and Children (WIC)–participating infants and children from birth to 3 years in an analysis of the relationship between WIC benefit redemption and continued WIC participation, 2019-2023 (n=188,368) **eTable 1.** Comparison of characteristics for included and excluded child WIC participants 0 to 3 years of age, their caregivers, and their households at certification in Southern California between Nov 2019 and June 2022 (certification periods=371,618) **eFigure 2.** Schematic for using benefit issuance (horizontal colored lines) in the determination of discontinued WIC participation (red squares) and continued WIC participation (blue circles) at the end of included certification periods for WIC-participating children in the study **eTable 2.** Association of category-specific household WIC benefit redemption with risk of discontinuation of WIC participation among child WIC participants 0 to 3 years of age at eligibility certification in Southern California in November, 2019 to June, 2022 (n, certification periods=366,212) **eTable 3.** Association of category-specific household WIC benefit redemption with risk of discontinuation of WIC participation by participant category among child WIC participants 0 to 3 years of age at eligibility certification in Southern California in November, 2019 to June, 2022 (n, certification periods=366,212) **eTable 4.** Association of category-specific household WIC benefit redemption with risk of discontinuation of WIC participation compared to 70 to 100% redemption among child WIC participants 0 to 3 years of age at eligibility certification in Southern California in November, 2019 to June, 2022 (n, certification periods=366,212), excluding % total benefit redemption as a covariate

Supplement 2.
Data Sharing Statement
