# Supplementary material for: Redemption of Specific Categories of WIC Food Benefits and Risk of Program Discontinuation
Source: JAMA Netw Open. 2025 Dec 3;8(12):e2546544. doi: 10.1001/jamanetworkopen.2025.46544 (PMC12676361; doi:10.1001/jamanetworkopen.2025.46544)
Supplement: Supplement 1. — eMethods. eFigure 1. Flow chart for inclusion of Special Supplemental Nutrition Program for Women, Infants, and Children (WIC)–participating infants and children from birth to 3 years in an analysis of the relationship between WIC benefit redemption and continued WIC participation, 2019-2023 (n=188,368) eTable 1. Comparison of characteristics for included and excluded child WIC participants 0 to 3 years of age, their caregivers, and their households at certification in Southern California between Nov 2019 and June 2022 (certification periods=371,618) eFigure 2. Schematic for using benefit issuance (horizontal colored lines) in the determination of discontinued WIC participation (red squares) and continued WIC participation (blue circles) at the end of included certification periods for WIC-participating children in the study eTable 2. Association of category-specific household WIC benefit redemption with risk of discontinuation of WIC participation among child WIC participants 0 to 3 years of age at eligibility certification in Southern California in November, 2019 to June, 2022 (n, certification periods=366,212) eTable 3. Association of category-specific household WIC benefit redemption with risk of discontinuation of WIC participation by participant category among child WIC participants 0 to 3 years of age at eligibility certification in Southern California in November, 2019 to June, 2022 (n, certification periods=366,212) eTable 4. Association of category-specific household WIC benefit redemption with risk of discontinuation of WIC participation compared to 70 to 100% redemption among child WIC participants 0 to 3 years of age at eligibility certification in Southern California in November, 2019 to June, 2022 (n, certification periods=366,212), excluding % total benefit redemption as a covariate [file jamanetwopen-e2546544-s001.pdf]

Supplemental Online Content

Chaparro MP, Whaley SE, Anderson CE. Redemption of specific categories of WIC food benefits and risk of program discontinuation. *JAMA Netw Open*. 2025;8(12):e2546544. doi:10.1001/jamanetworkopen.2025.46544

eMethods.
eFigure 1. Flow chart for inclusion of Special Supplemental Nutrition Program for Women, Infants, and Children (WIC)-participating infants and children ages 0-3 years in an analysis of the relationship between WIC benefit redemption and continued WIC participation, 2019-2023 (n=188,368)
eTable 1. Comparison of characteristics for included and excluded child WIC participants 0 to 3 years of age, their caregivers, and their households at certification in Southern California between Nov 2019 and June 2022 (certification periods=371,618)
eFigure 2. Schematic for using benefit issuance (horizontal colored lines) in the determination of discontinued WIC participation (red squares) and continued WIC participation (blue circles) at the end of included certification periods for WIC-participating children in the study
eTable 2. Association of category-specific household WIC benefit redemption with risk of discontinuation of WIC participation among child WIC participants 0 to 3 years of age at eligibility certification in Southern California in November, 2019 to June, 2022 (n, certification periods=366,212)
eTable 3. Association of category-specific household WIC benefit redemption with risk of discontinuation of WIC participation by participant category among child WIC participants 0 to 3 years of age at eligibility certification in Southern California in November, 2019 to June, 2022 (n, certification periods=366,212).
eTable 4. Association of category-specific household WIC benefit redemption with risk of discontinuation of WIC participation compared to 70 to 100% redemption among child WIC participants 0 to 3 years of age at eligibility certification in Southern California in November, 2019 to June, 2022 (n, certification periods=366,212), excluding % total benefit redemption as a covariate

This supplemental material has been provided by the authors to give readers additional information about their work.

© 2025 Chaparro MP et al. *JAMA Network Open.*

eMethods.

Study population

Exclusion criteria. To ensure that adequate redemption data were available to stably characterize redemption across the certification period and to align with previous analyses of WIC benefit redemption,[7,12] certification periods with missing issuance in the first month, missing issuance for 5 or more months in the entire period, missing issuance in 3 or more in the last 6 months of the period, or missing issuance in 2 or more months in the last 3 months of the period were excluded from analysis (**eFigure 1**).

Study measures

Discontinued WIC participation. Children with discontinued WIC participation at the end of the certification period could have a subsequent certification period included in the data if they were subsequently certified at 3 or more months after the end of the index certification period. An example of this is shown in **eFigure 2**. Briefly, the figure shows periods of certification for specific hypothetical children with horizontal lines. Gaps in these lines indicate periods between certifications- gaps of 2 or more months mean that the preceding certification period ended in discontinued WIC participation, indicated with a red square, while gaps of <2 months mean that the preceding certification period ended in continued WIC participation, indicated with a blue circle. As an example, child 1 had an initial certification in January 2020 (and remained certified through December 2020), after which point there was a 3 month gap in certification (meaning that the first observed certification period ended in discontinued WIC participation, indicated with a red square). The child subsequently was certified on WIC in April 2021 (remaining certified through March 2022), and there was no gap in certification for the next certification period (beginning in April 2022) meaning the second certification period ended in continued WIC participation. Each individual WIC participant contributed an observation to the dataset for each 12-month certification period during the study. Households with multiple WIC participants participating simultaneously contributed one observation to the dataset for every individual for each of that individual's observed certification periods.

Race and ethnicity. Child race/ethnicity were reported by the child's caregiver from a list with specified options, including: American Indian/Alaska Native, Asian Indian, Black or African American, Cambodian, Chinese, Fijian, Filipino, Guamanian or Chamorro, Hmong, Japanese, Korean, Laotian, Multiple races, Native Hawaiian, Other (not specified), Samoan, Thai, Tongan, Unknown, Vietnamese, and White. For analysis, the following groups were collapsed into 1) Non-Hispanic (NH) Asian: Asian Indian, Cambodian, Chinese, Fijian, Filipino, Guamanian or Chamorro, Hmong, Japanese, Korean, Laotian, Native Hawaiian, Samoan, Thai, Tongan, Vietnamese. 2) NH Black: Black or African American. 3) NH Other: American Indian/Alaska Native, Multiple, Other (not specified).

COVID-19 related covariates. During the COVID-19 pandemic and the infant formula shortage crisis, WIC implemented several changes which occurred in the middle of our study period (November 2019 – June 2023). These changes included an augmented fruit and vegetable benefit, introduced in June 2021 and available through June 2023; brand and package size flexibilities, introduced in May 2020 and available through April 2021; and WIC infant formula flexibilities, introduced in April 2022 and available through February 2023. For this reason, we included as covariates the number of months (0-12 months) in the certification period that the household was exposed to each of these three changes.

© 2025 Chaparro MP et al. *JAMA Network Open.*

eFigure 1. Flow chart for inclusion of Special Supplemental Nutrition Program for Women, Infants, and Children (WIC)-participating infants and children ages 0-3 years in an analysis of the relationship between WIC benefit redemption and continued WIC participation, 2019-2023 (n=188,368).

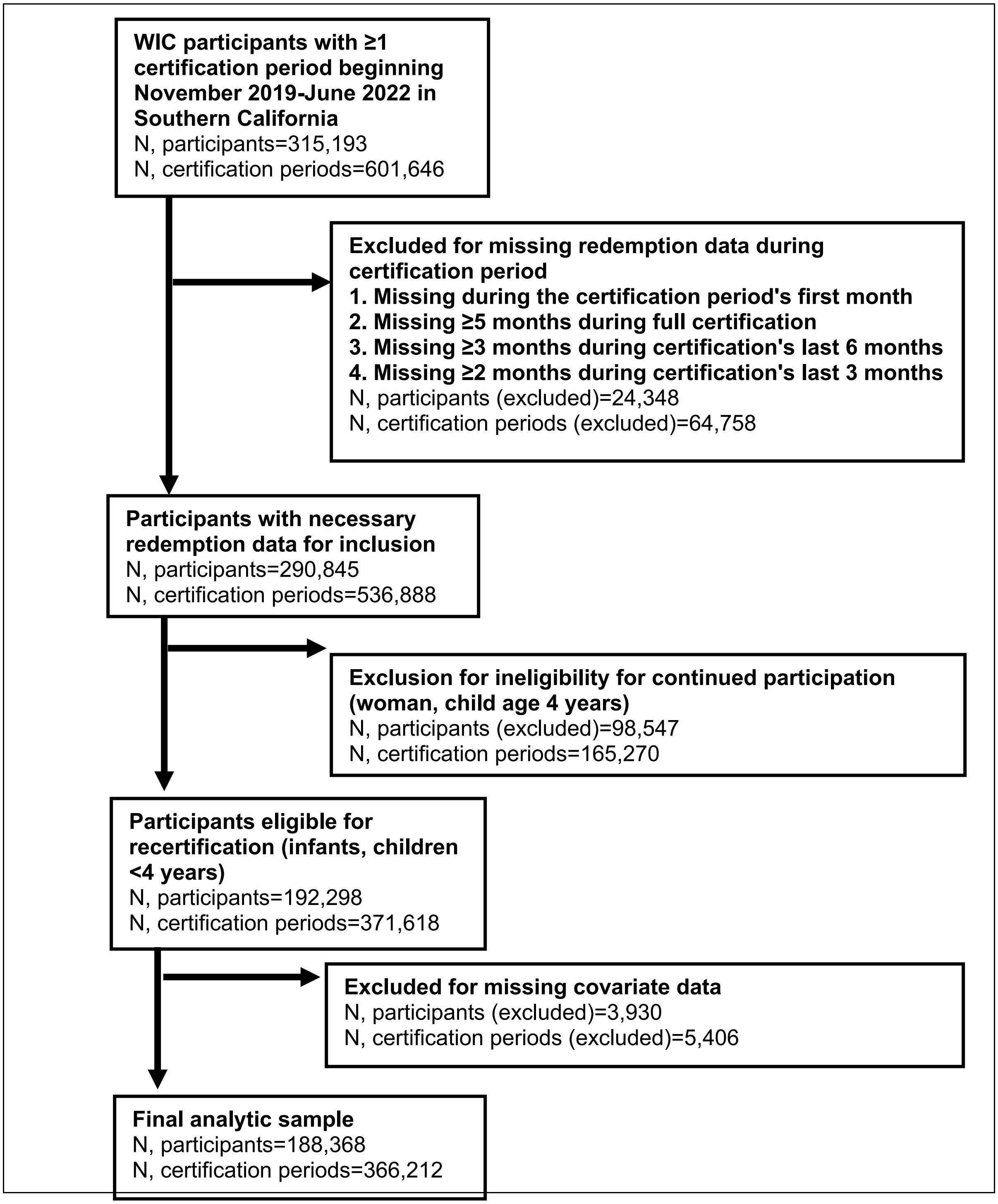

© 2025 Chaparro MP et al. *JAMA Network Open.*

eTable 1. Comparison of characteristics for included and excluded child WIC participants 0 to 3 years of age, their caregivers, and their households at certification in Southern California between Nov 2019 and June 2022 (certification periods=371,618).

Variable		Included N=366,212	Excluded N=5,406
**Child**			
Participant category			
	Formula fed infant	55190 (15.1)	990 (18.3)
	Partially breastfed infant	32070 (8.8)	531 (9.8)
	Fully breastfed infant	19057 (5.2)	268 (5.0)
	Child 1-2 years	87534 (23.9)	1321 (24.4)
	Child 2-3 years	87720 (24.0)	1194 (22.1)
	Child 3-4 years	84641 (23.1)	1102 (20.4)
Male		185968 (50.8)	2761 (51.1)
Race/ethnicity-language			
	non-Hispanic Asian, EN	9602 (2.6)	119 (2.2)
	non-Hispanic Asian, OT	14811 (4.0)	100 (1.8)
	non-Hispanic Black	20812 (5.7)	423 (7.8)
	Hispanic, EN	208482 (56.9)	3043 (56.3)
	Hispanic, SP	88837 (24.3)	1255 (23.2)
	non-Hispanic White	10897 (3.0)	240 (4.4)
	non-Hispanic Other	12771 (3.5)	226 (4.2)
**Caregiver/household**			
Caregiver education			
	<High school	87966 (24.0)	1566 (29.0)
	Completed high school	161563 (44.1)	2352 (43.5)
	>High school	116683 (31.9)	1488 (27.5)
Medicaid participation		311339 (85.0)	3905 (72.2)
SNAP participation		134147 (36.6)	1845 (34.1)
Household income			
	<50% FPL	99963 (27.3)	1925 (35.6)
	50 to 100% FPL	136156 (37.2)	1831 (33.9)
	>100% FPL	130093 (35.5)	1650 (30.5)
Household size			
	<4 people	141978 (38.8)	2416 (44.7)
	4-5 people	176866 (48.3)	2354 (43.5)
	≥6 people	47368 (12.9)	636 (11.8)
Family members on WIC			
	1	150152 (41.0)	2191 (40.5)
	2	144040 (39.3)	2208 (40.8)
	≥3	72020 (19.7)	1007 (18.6)
Months of certification during…, mean ± SD			
	CVB augment	7.2 ± 5.1	3.9 ± 4.9
	Brand/package size flexibilities	4.7 ± 4.7	7.0 ± 4.1
	Infant formula flexibilities	3.0 ± 4.0	1.4 ± 3.1

Abbreviations: CVB, Cash Value Benefit; EN, English-speaking; FPL, federal poverty level; OT, Other language-speaking; rep., representative; SNAP, Supplemental Nutrition Assistance Program; SD, standard deviation; SP, Spanish-speaking; WIC, Special Supplemental Nutrition Program for Women, Infants, and Children.

© 2025 Chaparro MP et al. *JAMA Network Open.*

eFigure 2. Schematic for using benefit issuance (horizontal colored lines) in the determination of discontinued WIC participation (red squares) and continued WIC participation (blue circles) at the end of included certification periods for WIC-participating children in the study.

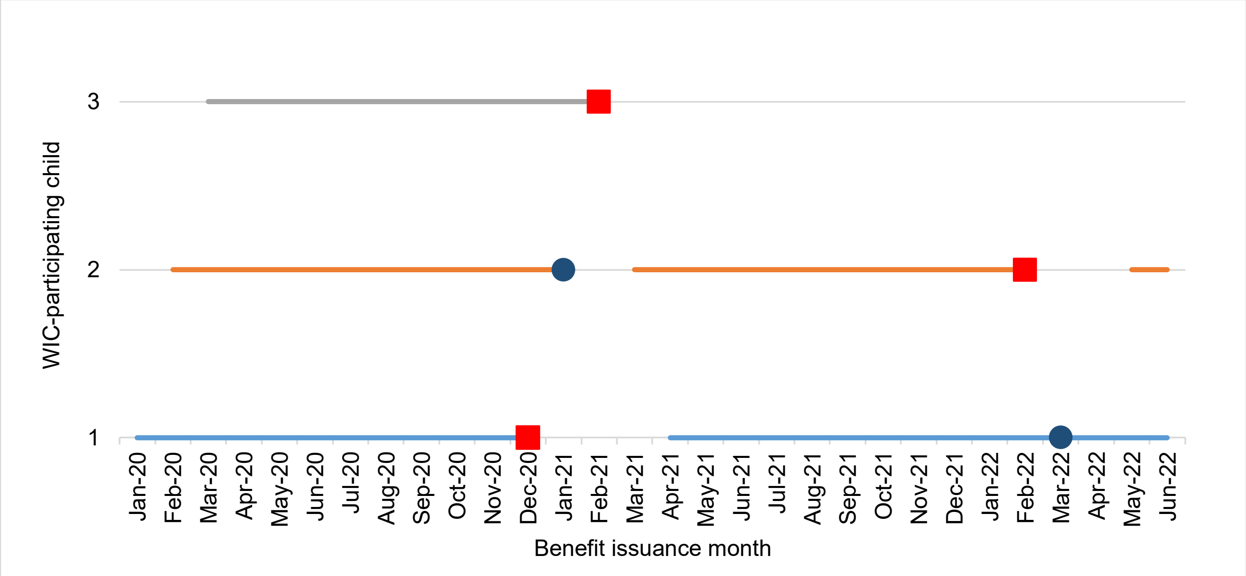

© 2025 Chaparro MP et al. *JAMA Network Open.*

eTable 2. Association of category-specific household WIC benefit redemption with risk of discontinuation of WIC participation among child WIC participants 0 to 3 years of age at eligibility certification in Southern California in November, 2019 to June, 2022 (n, certification periods=366,212).[a]

	Redemption percentage		
	Dichotomous		Ordinal
Category	<70%<br>RR (95% CI)	70 to 100%<br>RR (95% CI)	1-category decrease<br>RR (95% CI)
FV	1.35 (1.33, 1.37)*	1.00 (ref)	1.10 (1.10, 1.11)*
Eggs	1.15 (1.13, 1.17)*	1.00 (ref)	1.07 (1.06, 1.07)*
Whole milk	1.25 (1.22, 1.29)*	1.00 (ref)	1.07 (1.07, 1.08)*
Cheese/tofu	0.97 (0.95, 0.99)*	1.00 (ref)	1.02 (1.01, 1.02)*
100% Juice	0.92 (0.90, 0.93)*	1.00 (ref)	1.00 (1.00, 1.01)
Reduced fat milk	0.98 (0.96, <1.00)*	1.00 (ref)	1.01 (>1.00, 1.01)*
Legumes	0.83 (0.82, 0.85)*	1.00 (ref)	0.96 (0.96, 0.97)*
Breakfast cereal	0.84 (0.83, 0.86)*	1.00 (ref)	0.96 (0.96, 0.97)*
Bread/whole grain	0.85 (0.83, 0.86)*	1.00 (ref)	0.97 (0.97, 0.98)*
Yogurt	0.82 (0.80, 0.84)*	1.00 (ref)	0.95 (0.95, 0.96)*
Infant formula	1.18 (1.15, 1.22)*	1.00 (ref)	1.03 (1.02, 1.03)*
Therapeutic formula	1.01 (0.90, 1.13)	1.00 (ref)	0.99 (0.97, 1.01)
Infant FV	0.96 (0.93, 0.99)*	1.00 (ref)	1.00 (1.00, 1.01)
Infant cereal	0.86 (0.83, 0.89)*	1.00 (ref)	0.98 (0.97, 0.99)*
Infant meat	0.80 (0.71, 0.90)*	1.00 (ref)	0.96 (0.95, 0.98)*

Abbreviations: FV, fruits and vegetables; WIC, the Special Supplemental Nutrition Program for Women, Infants, and Children;
* Indicates values that are statistically significant at $p<0.05$.
[a] Associations are presented as risk ratio (95% Confidence interval), and were determined in multivariable generalized estimating equation Poisson regression models with an exchangeable correlation structure for (dependent variable) discontinuation of WIC participation, regressed on (independent variables) child sex, race/ethnicity-language preference, and age category; dichotomous or interval-scaled category-specific household benefit redemption and any months of missing issuance (yes, no); family representative educational attainment; and household Medicaid participation, SNAP participation, income category, size, the number of WIC participants, and the average total WIC food benefit redemption percentage (continuous) across the certification period; and the number of months during the certification period in which an augmented FV benefit was issued, the number of months in which brand/package size flexibility waivers were in place, and the number of months in which infant formula brand and package size flexibility waivers were in place. Models accommodated clustering of multiple certification periods for individual children and multiple children in families.

© 2025 Chaparro MP et al. *JAMA Network Open.*

eTable 3. Association of category-specific household WIC benefit redemption with risk of discontinuation of WIC participation by participant category among child WIC participants 0 to 3 years of age at eligibility certification in Southern California in November, 2019 to June, 2022 (n, certification periods=366,212).[a]

		Redemption percentage				
		Dichotomous			Ordinal	
		<70%	70 to 100%	*P* [b]	*1-category decrease*	*P* [c]
Food	Participant category	RR (95% CI)	RR (95% CI)		RR (95% CI)	
FV				<0.0001		<0.0001
	Formula fed infant	1.72 (1.65, 1.79)*	1.00 (ref)		1.15 (1.14, 1.16)*	
	Partial BF infant	1.91 (1.81, 2.02)*	1.00 (ref)		1.19 (1.18, 1.20)*	
	Fully BF infant	2.41 (2.24, 2.59)*	1.00 (ref)		1.23 (1.21, 1.24)*	
	Child, age 1 y	2.08 (2.02, 2.15)*	1.00 (ref)		1.18 (1.18, 1.19)*	
	Child, age 2 y	1.10 (1.08, 1.13)*	1.00 (ref)		1.06 (1.06, 1.07)*	
	Child, age 3 y	0.97 (0.95, 0.99)*	1.00 (ref)		1.04 (1.03, 1.04)*	
Eggs				<0.0001		<0.0001
	Formula fed infant	1.62 (1.55, 1.69)*	1.00 (ref)		1.12 (1.11, 1.13)*	
	Partial BF infant	1.66 (1.57, 1.76)*	1.00 (ref)		1.14 (1.13, 1.15)*	
	Fully BF infant	2.02 (1.87, 2.18)*	1.00 (ref)		1.17 (1.16, 1.19)*	
	Child, age 1 y	1.75 (1.69, 1.81)*	1.00 (ref)		1.15 (1.14, 1.15)*	
	Child, age 2 y	0.92 (0.89, 0.94)*	1.00 (ref)		1.03 (1.02, 1.03)*	
	Child, age 3 y	0.81 (0.79, 0.83)*	1.00 (ref)		1.00 (0.99, 1.01)	
Whole milk				<0.0001		<0.0001
	Formula fed infant	1.88 (1.77, 2.00)*	1.00 (ref)		1.15 (1.13, 1.16)*	
	Partial BF infant	1.96 (1.79, 2.15)*	1.00 (ref)		1.16 (1.14, 1.18)*	
	Fully BF infant	1.98 (1.74, 2.25)*	1.00 (ref)		1.16 (1.14, 1.18)*	
	Child, age 1 y	1.31 (1.26, 1.36)*	1.00 (ref)		1.07 (1.07, 1.08)*	
	Child, age 2 y	0.95 (0.89, 1.01)	1.00 (ref)		1.01 (1.00, 1.03)	
	Child, age 3 y	0.76 (0.72, 0.79)*	1.00 (ref)		0.97 (0.96, 0.98)*	
Cheese/tofu				<0.0001		<0.0001
	Formula fed infant	1.38 (1.32, 1.44)*	1.00 (ref)		1.07 (1.06, 1.07)*	
	Partial BF infant	1.36 (1.29, 1.45)*	1.00 (ref)		1.07 (1.06, 1.09)*	
	Fully BF infant	1.51 (1.39, 1.64)*	1.00 (ref)		1.10 (1.09, 1.11)*	
	Child, age 1 y	1.45 (1.40, 1.50)*	1.00 (ref)		1.09 (1.08, 1.09)*	
	Child, age 2 y	0.78 (0.76, 0.80)*	1.00 (ref)		0.98 (0.97, 0.98)*	
	Child, age 3 y	0.69 (0.67, 0.71)*	1.00 (ref)		0.95 (0.95, 0.96)*	
100% Juice				<0.0001		<0.0001
	Formula fed infant	1.39 (1.32, 1.46)*	1.00 (ref)		1.07 (1.06, 1.07)*	
	Partial BF infant	1.38 (1.30, 1.48)*	1.00 (ref)		1.07 (1.06, 1.09)*	
	Fully BF infant	1.51 (1.39, 1.65)*	1.00 (ref)		1.10 (1.08, 1.11)*	
	Child, age 1 y	1.36 (1.32, 1.41)*	1.00 (ref)		1.08 (1.07, 1.08)*	
	Child, age 2 y	0.74 (0.72, 0.76)*	1.00 (ref)		0.97 (0.96, 0.97)*	
	Child, age 3 y	0.66 (0.65, 0.68)*	1.00 (ref)		0.95 (0.94, 0.95)*	
Reduced fat milk				<0.0001		<0.0001
	Formula fed infant	1.42 (1.35, 1.49)*	1.00 (ref)		1.05 (1.05, 1.06)*	
	Partial BF infant	1.52 (1.42, 1.62)*	1.00 (ref)		1.07 (1.06, 1.08)*	
	Fully BF infant	1.63 (1.48, 1.80)*	1.00 (ref)		1.09 (1.08, 1.11)*	
	Child, age 1 y	1.59 (1.52, 1.67)*	1.00 (ref)		1.08 (1.07, 1.09)*	
	Child, age 2 y	0.83 (0.80, 0.85)*	1.00 (ref)		0.97 (0.97, 0.98)*	
	Child, age 3 y	0.72 (0.70, 0.74)*	1.00 (ref)		0.95 (0.94, 0.95)*	
Legumes				<0.0001		<0.0001
	Formula fed infant	1.20 (1.14, 1.26)*	1.00 (ref)		1.02 (1.01, 1.03)*	
	Partial BF infant	1.23 (1.15, 1.31)*	1.00 (ref)		1.02 (1.01, 1.03)*	

© 2025 Chaparro MP et al. *JAMA Network Open.*

Fully BF infant	1.36 (1.24, 1.50)*	1.00 (ref)		1.04 (1.03, 1.06)*	
Child, age 1 y	1.21 (1.16, 1.26)*	1.00 (ref)		1.03 (1.02, 1.03)*	
Child, age 2 y	0.66 (0.64, 0.68)*	1.00 (ref)		0.92 (0.92, 0.93)*	
Child, age 3 y	0.60 (0.58, 0.62)*	1.00 (ref)		0.90 (0.89, 0.90)*	
Breakfast cereal			<0.0001		<0.0001
Formula fed infant	1.22 (1.16, 1.27)*	1.00 (ref)		1.02 (1.01, 1.03)*	
Partial BF infant	1.19 (1.12, 1.27)*	1.00 (ref)		1.02 (1.01, 1.03)*	
Fully BF infant	1.37 (1.25, 1.50)*	1.00 (ref)		1.04 (1.03, 1.06)*	
Child, age 1 y	1.24 (1.19, 1.29)*	1.00 (ref)		1.03 (1.02, 1.03)*	
Child, age 2 y	0.68 (0.66, 0.70)*	1.00 (ref)		0.92 (0.92, 0.93)*	
Child, age 3 y	0.61 (0.59, 0.63)*	1.00 (ref)		0.90 (0.90, 0.91)*	
Bread/whole grain			<0.0001		<0.0001
Formula fed infant	1.30 (1.24, 1.37)*	1.00 (ref)		1.04 (1.03, 1.05)*	
Partial BF infant	1.29 (1.20, 1.37)*	1.00 (ref)		1.03 (1.02, 1.04)*	
Fully BF infant	1.37 (1.26, 1.49)*	1.00 (ref)		1.05 (1.04, 1.06)*	
Child, age 1 y	1.23 (1.18, 1.28)*	1.00 (ref)		1.02 (1.02, 1.03)*	
Child, age 2 y	0.67 (0.65, 0.69)*	1.00 (ref)		0.92 (0.91, 0.92)*	
Child, age 3 y	0.61 (0.59, 0.62)*	1.00 (ref)		0.90 (0.90, 0.91)*	
Yogurt			<0.0001		<0.0001
Formula fed infant	1.17 (1.12, 1.23)*	1.00 (ref)		1.01 (1.00, 1.02)*	
Partial BF infant	1.16 (1.09, 1.23)*	1.00 (ref)		1.01 (1.00, 1.02)*	
Fully BF infant	1.23 (1.13, 1.35)*	1.00 (ref)		1.02 (1.01, 1.04)*	
Child, age 1 y	1.14 (1.09, 1.19)*	1.00 (ref)		1.01 (1.00, 1.01)	
Child, age 2 y	0.69 (0.67, 0.71)*	1.00 (ref)		0.92 (0.91, 0.92)*	
Child, age 3 y	0.61 (0.60, 0.63)*	1.00 (ref)		0.90 (0.89, 0.90)*	
Infant formula			<0.0001		<0.0001
Formula fed infant	1.50 (1.44, 1.56)*	1.00 (ref)		1.07 (1.06, 1.08)*	
Partial BF infant	1.42 (1.34, 1.50)*	1.00 (ref)		1.06 (1.05, 1.07)*	
Fully BF infant	1.40 (1.25, 1.56)*	1.00 (ref)		1.04 (1.03, 1.06)*	
Child, age 1 y	1.13 (1.06, 1.21)*	1.00 (ref)		1.04 (1.03, 1.05)*	
Child, age 2 y	0.91 (0.85, 0.97)*	1.00 (ref)		0.98 (0.96, 0.99)*	
Child, age 3 y	0.87 (0.81, 0.92)*	1.00 (ref)		0.97 (0.95, 0.98)*	
Therapeutic formula			0.06		0.19
Formula fed infant	0.99 (0.84, 1.16)	1.00 (ref)		0.99 (0.96, 1.03)	
Partial BF infant	1.26 (1.02, 1.55)*	1.00 (ref)		1.03 (0.99, 1.07)	
Fully BF infant	1.18 (0.78, 1.76)	1.00 (ref)		1.04 (0.95, 1.14)	
Child, age 1 y	1.11 (0.86, 1.43)	1.00 (ref)		1.00 (0.96, 1.05)	
Child, age 2 y	0.73 (0.53, 1.00)*	1.00 (ref)		0.96 (0.91, 1.02)	
Child, age 3 y	0.89 (0.66, 1.19)	1.00 (ref)		0.93 (0.87, 1.00)	
Infant FV			<0.0001		<0.0001
Formula fed infant	1.17 (1.11, 1.23)*	1.00 (ref)		1.04 (1.03, 1.05)*	
Partial BF infant	1.29 (1.20, 1.38)*	1.00 (ref)		1.06 (1.05, 1.08)*	
Fully BF infant	1.26 (1.13, 1.40)*	1.00 (ref)		1.06 (1.05, 1.08)*	
Child, age 1 y	0.90 (0.84, 0.96)*	1.00 (ref)		1.00 (0.99, 1.01)	
Child, age 2 y	0.77 (0.72, 0.82)*	1.00 (ref)		0.95 (0.94, 0.96)*	
Child, age 3 y	0.69 (0.66, 0.73)*	1.00 (ref)		0.94 (0.93, 0.95)*	
Infant cereal			<0.0001		<0.0001
Formula fed infant	0.99 (0.94, 1.06)	1.00 (ref)		1.01 (1.00, 1.01)	
Partial BF infant	1.13 (1.05, 1.23)*	1.00 (ref)		1.03 (1.02, 1.04)	
Fully BF infant	1.05 (0.94, 1.18)	1.00 (ref)		1.03 (1.01, 1.04)	
Child, age 1 y	0.85 (0.79, 0.91)*	1.00 (ref)		0.99 (0.98, 1.00)	
Child, age 2 y	0.71 (0.66, 0.77)*	1.00 (ref)		0.95 (0.94, 0.96)*	
Child, age 3 y	0.65 (0.61, 0.69)*	1.00 (ref)		0.93 (0.92, 0.94)*	
Infant meats			0.0005		<0.0001
Formula fed infant	0.74 (0.32, 1.71)	1.00 (ref)		1.01 (0.89, 1.14)	
Partial BF infant	1.22 (0.80, 1.86)	1.00 (ref)		1.01 (0.97, 1.06)	

© 2025 Chaparro MP et al. *JAMA Network Open.*

Fully BF infant	1.02 (0.83, 1.25)	1.00 (ref)	1.01 (0.98, 1.04)
Child, age 1 y	0.93 (0.70, 1.23)	1.00 (ref)	1.00 (0.96, 1.04)
Child, age 2 y	0.68 (0.52, 0.88)*	1.00 (ref)	0.92 (0.89, 0.95)*
Child, age 3 y	0.56 (0.46, 0.68)*	1.00 (ref)	0.91 (0.89, 0.94)*

Abbreviations: BF, breastfed; FV, fruits and vegetables; SNAP, Supplemental Nutrition Assistance Program; WIC, the Special Supplemental Nutrition Program for Women, Infants, and Children;
* Indicates values that are statistically significant at p<0.05.
[a] Associations are presented as risk ratio (95% Confidence interval), and were determined in multivariable generalized estimating equation Poisson regression models with an exchangeable correlation structure for (dependent variable) discontinuation of WIC participation, regressed on (independent variables) child sex, race/ethnicity-language preference, and age category; dichotomous or ordinal interval-scaled category-specific household benefit redemption and any months of missing issuance (yes, no); family representative educational attainment; and household Medicaid participation, SNAP participation, income category, size, the number of WIC participants, and the average total WIC food benefit redemption percentage (continuous) across the certification period; the number of months during the certification period in which an augmented FV benefit was issued, the number of months in which brand/package size flexibility waivers were in place, and the number of months in which infant formula brand and package size flexibility waivers were in place; and a two-way interaction of participant category with dichotomous or ordinal interval-scaled category-specific benefit redemption. Models accommodated clustering of multiple certification periods for individual children and multiple children in families.
[b] P-value is for the type 3 test of the interaction between dichotomous category-specific benefit redemption and participant category.
[c] P-value is for the type 3 test of the interaction between dichotomous category-specific benefit redemption and participant category.

© 2025 Chaparro MP et al. *JAMA Network Open.*

eTable 4. Association of category-specific household WIC benefit redemption with risk of discontinuation of WIC participation compared to 70 to 100% redemption among child WIC participants 0 to 3 years of age at eligibility certification in Southern California in November, 2019 to June, 2022 (n, certification periods=366,212), excluding % total benefit redemption as a covariate. [a]

	Interval-scaled category-specific benefit redemption						
**Category**	**<10%**	**10 to <20%**	**20 to <30%**	**30 to <40%**	**40 to <50%**	**50 to <60%**	**60 to <70%**
FV	3.75 (3.69, 3.82)*	2.88 (2.81, 2.96)*	2.62 (2.55, 2.69)*	2.22 (2.16, 2.28)*	1.99 (1.94, 2.05)*	1.74 (1.70, 1.78)*	1.48 (1.45, 1.52)*
Eggs	3.44 (3.38, 3.50)*	2.69 (2.62, 2.76)*	2.33 (2.27, 2.39)*	2.11 (2.06, 2.17)*	1.88 (1.83, 1.94)*	1.59 (1.56, 1.63)*	1.45 (1.41, 1.48)*
Whole milk	3.86 (3.76, 3.96)*	2.80 (2.68, 2.93)*	2.52 (2.40, 2.64)*	2.03 (1.94, 2.12)*	2.05 (1.95, 2.15)*	1.69 (1.62, 1.77)*	1.47 (1.41, 1.54)*
Cheese/tofu	3.07 (3.02, 3.12)*	2.31 (2.26, 2.37)*	2.04 (1.99, 2.09)*	1.87 (1.83, 1.92)*	1.62 (1.58, 1.67)*	1.45 (1.42, 1.49)*	1.36 (1.33, 1.40)*
100% Juice	2.92 (2.87, 2.97)*	2.20 (2.15, 2.26)*	1.98 (1.94, 2.03)*	1.75 (1.71, 1.80)*	1.57 (1.52, 1.61)*	1.39 (1.36, 1.42)*	1.21 (1.19, 1.25)*
Reduced fat milk	2.71 (2.66, 2.75)*	1.97 (1.92, 2.02)*	1.82 (1.77, 1.87)*	1.63 (1.58, 1.67)*	1.52 (1.48, 1.56)*	1.39 (1.35, 1.42)*	1.24 (1.20, 1.27)*
Legumes	2.77 (2.72, 2.82)*	1.97 (1.92, 2.02)*	1.76 (1.71, 1.80)*	1.60 (1.56, 1.65)*	1.43 (1.39, 1.47)*	1.30 (1.27, 1.33)*	1.21 (1.18, 1.25)*
Breakfast cereal	2.80 (2.75, 2.85)*	2.03 (1.98, 2.08)*	1.76 (1.72, 1.80)*	1.58 (1.54, 1.63)*	1.46 (1.43, 1.50)*	1.33 (1.29, 1.36)*	1.22 (1.18, 1.25)*
Bread/whole grain	2.63 (2.58, 2.67)*	1.94 (1.89, 1.98)*	1.67 (1.63, 1.71)*	1.56 (1.52, 1.60)*	1.38 (1.34, 1.42)*	1.30 (1.27, 1.33)*	1.19 (1.15, 1.22)*
Yogurt	2.45 (2.40, 2.49)*	1.90 (1.85, 1.94)*	1.69 (1.65, 1.74)*	1.53 (1.49, 1.57)*	1.37 (1.33, 1.41)*	1.28 (1.24, 1.31)*	1.21 (1.18, 1.25)*
Infant formula	2.25 (2.16, 2.35)*	2.64 (2.44, 2.85)*	2.20 (2.05, 2.36)*	2.11 (1.97, 2.27)*	2.04 (1.90, 2.19)*	1.61 (1.53, 1.69)*	1.51 (1.43, 1.59)*
Therapeutic formula	1.27 (1.05, 1.53)*	1.18 (0.82, 1.72)	1.32 (0.92, 1.88)	1.54 (1.19, 2.00)*	1.46 (1.11, 1.92)*	1.27 (1.08, 1.50)*	1.19 (0.98, 1.44)
Infant FV	2.43 (2.35, 2.51)*	1.72 (1.64, 1.80)*	1.53 (1.45, 1.61)*	1.57 (1.49, 1.64)*	1.30 (1.22, 1.38)*	1.34 (1.27, 1.41)*	1.16 (1.10, 1.23)*
Infant cereal	2.21 (2.14, 2.29)*	1.61 (1.53, 1.68)*	1.41 (1.34, 1.49)*	1.43 (1.36, 1.50)*	1.27 (1.18, 1.37)*	1.30 (1.23, 1.37)*	1.13 (1.07, 1.19)*
Infant meat	1.88 (1.68, 2.10)*	1.27 (1.08, 1.50)*	1.49 (1.24, 1.79)*	1.22 (1.00, 1.49)*	1.43 (1.14, 1.79)*	1.15 (0.93, 1.42)*	0.90 (0.71, 1.15)

Abbreviations: FV, fruits and vegetables; WIC, the Special Supplemental Nutrition Program for Women, Infants, and Children;

* Indicates values that are statistically significant at p<0.05.

[a] Associations are presented as risk ratio (95% Confidence interval), and were determined in multivariable generalized estimating equation Poisson regression models for (dependent variable) discontinuation of WIC participation, regressed on (independent variables) child sex, race/ethnicity-language preference, and age category; interval-scaled category-specific household benefit redemption and any months of missing issuance (yes, no); family representative educational attainment; and household Medicaid participation, SNAP participation, income category, size, and the number of WIC participants; and the number of months during the certification period in which an augmented FV benefit was issued, the number of months in which brand/package size flexibility waivers were in place, and the number of months in which infant formula brand and package size flexibility waivers were in place. Models accommodated clustering of multiple certification periods for individual children and multiple children in families.

© 2025 Chaparro MP et al. *JAMA Network Open.*
