# Supplementary material for: Redemption of Specific Categories of WIC Food Benefits and Risk of Program Discontinuation
Source: JAMA Netw Open. 2025 Dec 3;8(12):e2546544. doi: 10.1001/jamanetworkopen.2025.46544 (PMC12676361; doi:10.1001/jamanetworkopen.2025.46544)
Supplement: Supplement 2. — Data Sharing Statement [file jamanetwopen-e2546544-s002.pdf]

Data Sharing Statement

Chaparro. Redemption of Specific Categories of WIC Food Benefits and Risk of Program Discontinuation. *JAMA Netw Open*. Published December 03, 2025. doi:10.1001/jamanetworkopen.2025.46544

Data
Data available: No

Additional Information
Explanation for why data not available: Data used in this study are confidential administrative data of the WIC program, and the data cannot be shared by the researchers due to a memorandum of understanding with the California WIC program.
